# PHF14 enhances DNA methylation of *SMAD7* gene to promote TGF-β-driven lung adenocarcinoma metastasis

**DOI:** 10.1038/s41421-023-00528-0

**Published:** 2023-04-18

**Authors:** Han Tian, Chenying Liu, Jianchen Yu, Jian Han, Jianan Du, Shujun Liang, Wenting Wang, Qin Liu, Rong Lian, Ting Zhu, Shanshan Wu, Tianyu Tao, Yaokai Ye, Jingjing Zhao, Yi Yang, Xun Zhu, Junchao Cai, Jueheng Wu, Mengfeng Li

**Affiliations:** 1 Zhongshan School of Medicine, Sun Yat-sen University, Guangzhou, Guangdong China; 2 Cancer Institute, Southern Medical University, Guangzhou, Guangdong China; 3 Department of Breast Pathology and Lab, Key Laboratory of Breast Cancer of Breast Cancer Prevention and Therapy, National Clinical Research Center of Cancer, Tianjin Medical University Cancer Institute and Hospital, Tianjin, China; 4 School of Chemistry, South China Normal University, Guangzhou, Guangdong China; 5 Department of Laboratory Medicine, Affiliated Cancer Hospital & Institute of Guangzhou Medical University, Guangzhou, Guangdong China; 6 Department of Biology, School of Basic Medical Science, Guangdong Medical University, Zhanjiang, Guangdong China; 7 Department of Gastrointestinal Surgery, The First Affiliated Hospital, Sun Yat-sen University, Guangzhou, Guangdong China; 8 Department of Cardiology, The First Affiliated Hospital, Sun Yat-sen University, Guangzhou, Guangdong China

**Keywords:** Metastasis, Cell signalling, Non-small-cell lung cancer

## Abstract

Aberrant activation of TGF-β signaling plays a pivotal role in cancer metastasis and progression. However, molecular mechanisms underlying the dysregulation of TGF-β pathway remain to be understood. Here, we found that SMAD7, a direct downstream transcriptional target and also a key antagonist of TGF-β signaling, is transcriptionally suppressed in lung adenocarcinoma (LAD) due to DNA hypermethylation. We further identified that PHF14 binds DNMT3B and serves as a DNA CpG motif reader, recruiting DNMT3B to the *SMAD7* gene locus, resulting in DNA methylation and transcriptional suppression of *SMAD7*. Our in vitro and in vivo experiments showed that PHF14 promotes metastasis through binding DNMT3B to suppress *SMAD7* expression. Moreover, our data revealed that PHF14 expression correlates with lowered SMAD7 level and shorter survival of LAD patients, and importantly that *SMAD7* methylation level of circulating tumor DNA (ctDNA) can potentially be used for prognosis prediction. Together, our present study illustrates a new epigenetic mechanism, mediated by PHF14 and DNMT3B, in the regulation of *SMAD7* transcription and TGF-β-driven LAD metastasis, and suggests potential opportunities for LAD prognosis.

## Introduction

Lung adenocarcinoma (LAD) is a major histological subtype of non-small cell lung carcinoma (NSCLC), which represents one of the most common malignant cancer types worldwide^1,2^. Due to the generally imperceptible symptoms at the early stages of LAD development, more than 50% of LAD patients are found with intrapulmonary or distant metastasis at their initial diagnosis, leading to approximately only 20% overall 5-year survival rate of LAD patients and less than 5% 5-year survival rate of patients with metastatic LAD^3^. Hence, identification of mechanisms regulating LAD metastasis, aiming at providing potential diagnostic and prognostic biomarkers as well as therapeutic targets for metastatic LAD, is key to improving the clinical outcome of the disease.

Accumulated evidence has revealed a pivotal role of TGF-β signaling in cancer metastasis^4^. The canonical TGF-β signaling cascade is initiated by binding of specific ligands to TGF-β receptors (TGFBR), which triggers oligomeric TGFBR1/2-mediated phosphorylation of the cytoplasmic effectors SMAD2/3 (receptor-regulated SMADs, R-SMADs). Subsequently, the phosphorylation-activated SMAD2/3 forms a heterotrimeric complex with SMAD4 (common SMAD, co-SMAD) and is transported to the nucleus, where the heterotrimeric complex interacts with other transcription factors/co-factors and mediates transcription of TGF-β target genes^5^. Notably, an antagonist of the TGF-β signaling, SMAD7 (also named inhibitory SMAD, I-SMAD), regulated at the transcriptional level by the R-SMADs/co-SMAD heterotrimeric complex, interacts with TGFBR1 and recruits SMURF2 or NEDD4L to promote poly-ubiquitination and proteasomal degradation of TGFBR1, eventually forming a negative feedback loop to maintain the physiological activity of TGF-β signaling^6,7^. Dysregulation of SMAD7 expression by a post-transcriptional regulation leads to aberrant activation of TGF-β signaling pathway, and such a scenario has been found in the malignant progression of multiple cancer types^8–12^. Interestingly, the mRNA level of *SMAD7* was found markedly lower in LAD samples than that in paired adjacent normal tissues, and lower *SMAD7* level correlated with poor prognosis of LAD patients^13–15^. In such a context, therefore, better understanding of the underlying mechanism of restraining *SMAD7* transcription in LAD may implicate promising therapeutic targets or prognostic biomarkers for metastatic LAD.

DNA methylation represents as a stable and inheritable epigenetic modification in eukaryotic cells, which mainly represses the process of transcription through disrupting the interaction between DNA elements and transcription factors^16^. The landscape of DNA methylation in cancers is considerably distorted, generally exhibiting hypomethylation in repeat-rich intergenic regions, resulting in genome instability, and hypermethylation in a subset of CpG-dense gene-associated regions, leading to transcriptional suppression of tumor suppressor genes^17^. Notably, DNMT3B, a de novo DNA methyltransferase, has been considered as an oncogene in cancer initiation and progression^18,19^. Numerous clinical studies have illustrated a great potential of DNA methylation as prognostic biomarkers and therapeutic targets for human cancers^20–22^. Nevertheless, the regulatory mechanism responsible for the role of DNMT3B in cancer malignancies remains largely unknown, and its diagnostic or prognostic effectiveness reflected in a large proportion of the registered clinical trials remains still suboptimal, suggesting that the mechanisms underlying de novo DNA methylation and the clinical significance of DNA methylation need to be further investigated and better understood.

In this current study, we find that a zinc finger protein PHF14 recruits DNMT3B to the *SMAD7* gene locus, resulting in *SMAD7* DNA methylation, transcriptional suppression and TGF-β signaling hyperactivition. In addition, we further demonstrate that PHF14 promotes LAD progression and metastasis through enhancing TGF-β signaling and illustrate that not only the *SMAD7* DNA methylation level in LAD tissues, but also that in circulating tumor DNA (ctDNA) of liquid biopsies correlate with poor prognosis of LAD patients.

## Results

### *SMAD7* expression is suppressed in LADs by DNA methylation

To understand the role of TGF-β signaling in lung adenocarcinoma progression, we initially found that the activity of TGF-β signaling, which is defined as the average of *TGFB1* and *SERPINE1* expression, significantly correlated with poor prognosis of LAD patients in the TCGA LUAD cohort (Supplementary Fig. S1a). Specifically, Kaplan-Meier analysis showed that LAD patients bearing high TGF-β activity in their lung tumors displayed a significantly shorter median survival time of 42.31 months and lower 5-year survival rate of 37.322%, in contrast to the 53.29-month median survival and 42.910% 5-year survival rate for those with low TGF-β activity (Supplementary Fig. S1a).

**Fig. 1:**
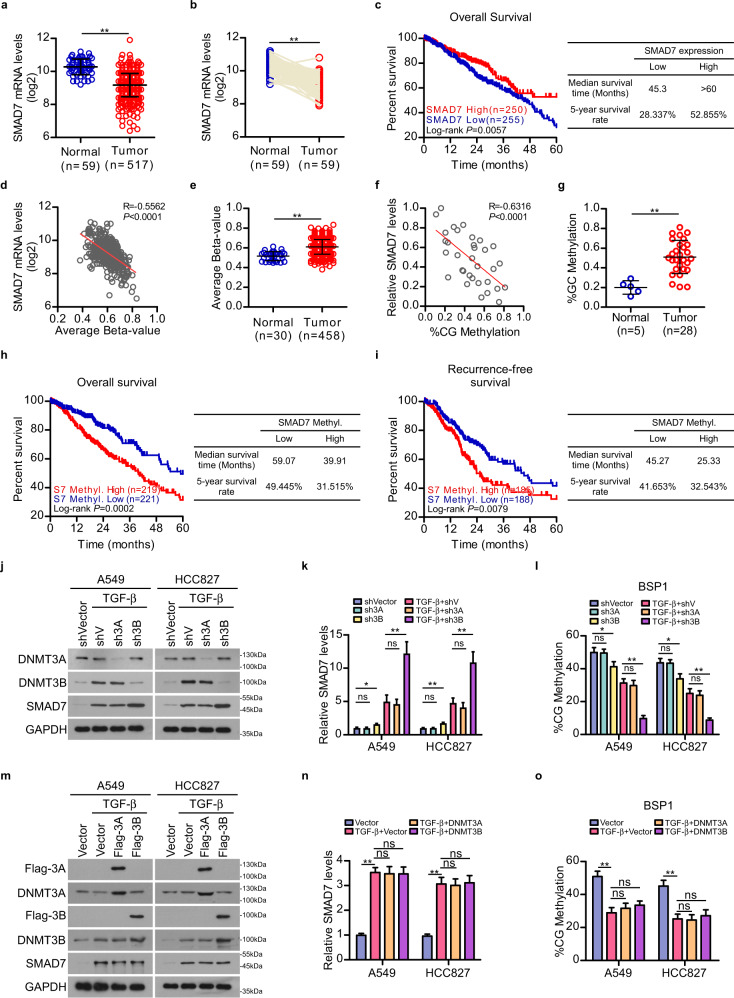
DNMT3B-catalyzed *SMAD7* DNA methylation correlates with poor prognosis of LAD patients. **a**, **b** Analysis of *SMAD7* expression in 517 cases of LAD tissue and 59 cases of adjacent normal lung tissue (**a**), and 59 pairs of human LAD tissue and adjacent normal lung tissue (**b**) in the TCGA LUAD datasets. **c** Kaplan-Meier analysis (Log-rank test) of the 5-year overall survival of LAD patients in the TCGA LUAD datasets, who were divided into low or high *SMAD7* expression subgroups. **d**, **e** Correlation between *SMAD7* mRNA levels and the average beta-values (DNA methylation level) of the selected 6 methylation probes in 460 cases of LAD tissues in the TCGA LUAD datasets (**d**), and analysis of the average beta-values of the 6 probes against LAD tissues and adjacent normal lung tissues in the TCGA LUAD datasets (**e**). **f**, **g** Correlation between *SMAD7* mRNA level and the average DNA methylation level of *SMAD7* in 5 cases of normal lung tissues and 28 cases of our collected LAD cohort (**f**), and analysis of the average DNA methylation level of *SMAD7* in adjacent normal lung tissues and LAD tissues in the cohort (**g**). **h**, **i** Kaplan-Meier analysis (Log-rank test) of the 5-year overall survival (left panel) and recurrence-free survival (right panel) of LAD patients in the TCGA LUAD datasets, who were divided into low or high *SMAD7* methylation level subgroups. **j**, **k** Western blotting (WB) and qPCR analyses showed the effect of silencing DNMT3A and DNMT3B on the protein and mRNA level of SMAD7 in A549 and HCC827 cells with indicated treatments. **l** Bisulfite-sequencing PCR (BSP) validated the DNA methylation levels of region 1 of *SMAD7* gene locus in A549 and HCC827 cells with indicated treatments. **m**, **n** WB and qPCR analysis showed the effect of overexpressing DNMT3A or DNMT3B on the protein and mRNA level of SMAD7 in A549 and HCC827 cells with indicated treatments. **o** BSP validated the DNA methylation level of region 1 of *SMAD7* gene locus in A549 and HCC827 cells with indicated treatments. For aforementioned WB, qPCR and BSP assays, cells were treated with TGF-β1 at final concentration of 5 ng/mL for 48 h before the indicated assays were performed (**j**–**o**). Error bars represent mean ± SD. Two-tailed unpaired Student’s *t*-test (**a**, **e** and **g**), two-tailed paired Student’s *t*-test (**b**), and two-way ANOVA multiple comparison analysis (**k**, **l**, **n**, and **o**), respectively, were used for statistical analysis. **P < 0.01; *P < 0.05; ns, not significant, *P* > 0.05.

The antagonist of TGF-β signaling, SMAD7, which is involved in the negative feed-back loop to counteract against TGF-β hyperactivation, is a direct transcriptional target of TGF-β signaling cascade (Supplementary Fig. S1b–d). The tumor suppressive role of SMAD7 was further validated in A549 and HCC827 LAD cell lines (Supplementary Fig. S1e–h). Of note, the expression of *SMAD7* was markedly suppressed in LAD as compared to the normal tissues (Fig. 1a, b), and high *SMAD7* expression level was associated with favorite prognosis of the LAD patients in the TCGA dataset (> 60 months median survival time and 52.855% 5-year survival rate in high *SMAD7* expression subgroup vs 45.3 months median survival time and 28.337% 5-year survival rate in low *SMAD7* expression subgroup, *P* = 0.0057) (Fig. 1c). Interestingly, *SMAD7* expression level was barely altered between LADs with higher TGF-β activity and LADs with lower TGF-β activity (Supplementary Fig. S1i), suggesting that despite the TGF-β signaling cascade mediated transcriptional activation of *SMAD7*, the transcriptional status of *SMAD7* might also be regulated by currently unidentified factors in LAD.

DNA methylation serves as an important epigenetic modification, which impedes gene transcription through altering chromosomal structure, DNA conformation and the interaction between regulatory DNA elements and protein factors. When the Shiny Methylation Analysis Resource Tool program was employed to identify the correlation of *SMAD7* DNA methylation level with *SMAD7* mRNA level and with the prognosis of LAD patients in the TCGA LUAD cohort^23^, among the 26 methylation probes distributed on *SMAD7* gene locus in the Illumina Infinium Human Methylation 450 K BeadChip, a panel of 6 probes was used as a probe set in the following analysis process, which detects sequences located on the 3′ UTR and CDS region of *SMAD7* gene, and methylation values of the 6 CpG sites were found to inversely correlate with *SMAD7* expression individually or aggregately in the TCGA LUAD dataset (Fig. 1d; Supplementary Figs. S1j, S2a and Table S1). Moreover, we found that the methylation level of *SMAD7* was elevated in LAD samples as compared to their paired adjacent normal controls (Fig. 1e). Analogously, the DNA methylation level of *SMAD7* was markedly increased in LAD tissues as compared to the normal lung tissues and negatively correlated with *SMAD7* mRNA levels in our collected tumor tissue cohort (Fig. 1f, g). In addition, high methylation levels of *SMAD7* were associated with poor prognosis (overall survival and recurrence-free survival) of LAD patients in TCGA LUAD dataset (Fig. 1h, i; Supplementary Fig. S2b). These data suggested that suppression of *SMAD7* expression can be induced by DNA hypermethylation, and that importantly, *SMAD7* DNA methylation levels revealed by the 6 probes predict unfavorable prognosis of LAD patients.

In the light that two de novo DNA methyltransferases DNMT3A and DNMT3B play distinct roles in lung cancer, as DNMT3B was implicated in promoting lung cancer by silencing tumor-suppressor genes, whereas DNMT3A suppressed *KRAS*^*G12D*^-mediated lung cancer progression^24–26^, we further investigated whether the oncogenic DNMT3B was involved in catalyzing de novo DNA hypermethylation of *SMAD7* gene. When DNMT3A or DNMT3B was silenced or ectopically overexpressed in A549 and HCC827 LAD cells with TGF-β treatment, only silencing DNMT3B significantly promoted *SMAD7* expression and diminished the DNA methylation of *SMAD7*, which could be partially reversed by additional ectopic overexpression of DNMT3B (Fig. 1j–l; Supplementary Fig. S2c–f). Whereas, in cells without TGF-β treatment, silencing DNMT3B markedly reduced *SMAD7* DNA methylation, while the mRNA level of *SMAD7* was slightly elevated (Fig. 1j–l; Supplementary Fig. S2c–f). In contrast, silencing or further overexpressing DNMT3A barely affected *SMAD7* DNA methylation and gene expression in cells with or without TGF-β treatment (Fig. 1j–l; Supplementary Fig. S2c–f). Interestingly, abundant DNMT3A or DNMT3B did not enhance *SMAD7* DNA methylation and had no effect on *SMAD7* transcription in cells with TGF-β treatment (Fig. 1m–o; Supplementary Fig. S2g), together suggesting that DNMT3B is the one de novo DNA methyltransferase that is required but seems to be insufficient for *SMAD7* DNA methylation and transcriptional suppression; therefore the detailed mechanisms for abrogation of the TGF-β-mediated *SMAD7* transcription in LAD, which may through facilitating the function of DNMT3B, still need to be further understood.

### PHF14 is a binding partner of DNMT3B

**Fig. 2:**
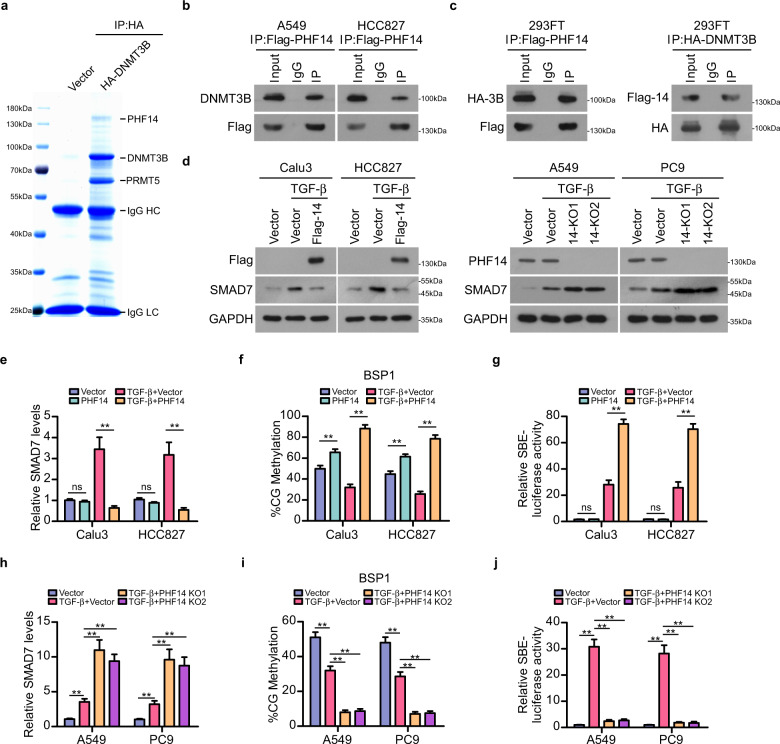
PHF14 is identified as a novel binding partner of DNMT3B. **a** Putative bound proteins of DNMT3B were analyzed by IP-MS assay. Representative image was derived from 3 independent experiments. **b**, **c** Immunoprecipitation assays with Flag-tagged PHF14 and HA-tagged DNMT3B, respectively, in LAD cells and 293FT cells validated an interaction between PHF14 and DNMT3B. Incubation of IgG-conjugated beads with cell lysates was employed as the negative control in these assays. Representative blots were derived from 3 independent experiments. **d** WB analysis showed the effect of overexpressing (left panel) or knocking out PHF14 (right panel) on the protein level of SMAD7 in indicated LAD cells. **e** qPCR analysis showed the effect of overexpressing PHF14 on the mRNA level of *SMAD7* in Calu3 and HCC827 cells with indicated treatments. **f** BSP validated the DNA methylation level of region 1 of *SMAD7* gene locus in Calu3 and HCC827 cells with indicated treatments. **g** Measurement of SBE-luciferase activity showed relative TGF-β signaling activates in Calu3 and HCC827 cells with indicated treatments. **h** qPCR analysis showed the effect of knocking out PHF14 on the mRNA level of *SMAD7* in A549 and PC9 cells with indicated treatments. **i** BSP validated the DNA methylation level of region 1 of *SMAD7* gene locus in A549 and PC9 cells with indicated treatments. **j** Measurement of SBE-luciferase activity showed relative TGF-β signaling activates in A549 and PC9 cells with indicated treatments. For aforementioned WB, qPCR, BSP and SBE-luciferase activity assays, cells were treated with TGF-β1 at final concentration of 5 ng/mL for 48 h before the indicated assays were performed (**d**–**j**). Error bars represent means ± SD derived from three independent experiments. Two-way ANOVA multiple comparison analysis was used for statistical analysis. **P < 0.01; ns, not significant, *P* > 0.05.

To gain an insight into the mechanism of DNMT3B-mediated abnormal DNA methylation on *SMAD7* locus in LAD, we further examined whether previously unidentified factor might regulate DNMT3B-mediated *SMAD7* DNA methylation and transcriptional suppression in LAD. The immunoprecipitation-mass spectrometry (IP-MS) assay was employed to screen uncharted binding partners of DNMT3B in A549 cell. Among the top-ranked putative binding proteins of DNMT3B, a plant homeodomain (PHD) finger-containing protein PHF14, which was recognized as an epigenetic suppressor, was identified to interact with DNMT3B (Supplementary Table S2), and the interaction was further validated by endogenous and exogenous co-IPs using LAD cells and 293FT cell (Fig. 2a–c; Supplementary Fig. S3a). Notably, the interaction between DNMT3B and PHF14 was moderately enhanced with TGF-β treatment (Supplementary Fig. S3a). Moreover, overexpressing PHF14 in LAD cells enhanced TGF-β-induced SMAD binding element (SBE)-luciferase activity with restrained *SMAD7* expression by facilitating *SMAD7* DNA methylation, while in cells without TGF-β treatment, the *SMAD7* DNA methylation level was moderately enhanced and the *SMAD7* mRNA level and TGF-β signaling activity were not markedly affected by ectopically overexpressed PHF14 (Fig. 2d–g; Supplementary Fig. S3b). In contrast, knocking out PHF14 in LAD cells impaired the TGF-β-induced SBE-luciferase activity by elevating *SMAD7* expression level with depleted *SMAD7* DNA methylation (Fig. 2d, h–j; Supplementary Fig. S3c). These results indicated that PHF14 is a newly identified binding partner of DNMT3B, which may promote TGF-β signaling activation through facilitating DNMT3B-mediated *SMAD7* DNA hypermethylation and transcriptional suppression in LAD.

### PHF14 facilities DNMT3B-mediated DNA methylation of *SMAD7*

**Fig. 3:**
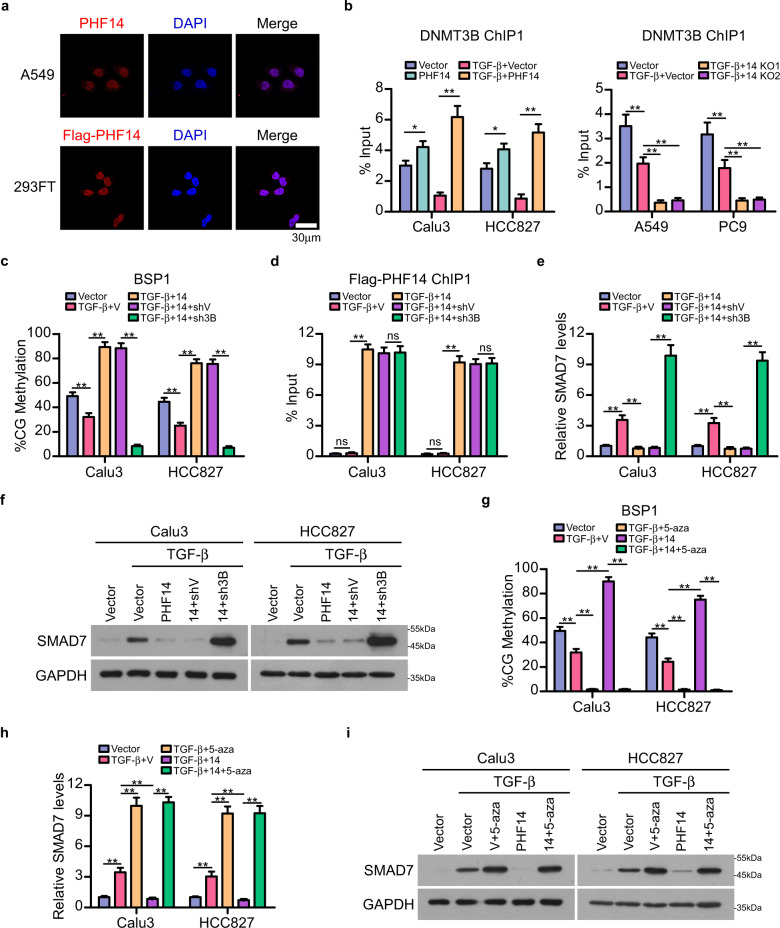
PHF14 facilities DNMT3B-mediated DNA methylation of *SMAD7*. **a** Representative images of subcellular localization of endogenous PHF14 in A549 cells and Flag-tagged PHF14 in 293FT cells (five random fields of view per slice, scale bar: 30 μm). **b** ChIP-qPCR assays validated the effect of overexpressing PHF14 (left) or knocking out PHF14 (right) on the occupancy of DNMT3B on region 1 of *SMAD7* gene locus in indicated cells. **c** BSP validated the DNA methylation level of region 1 of *SMAD7* gene locus in Calu3 and HCC827 cells with indicated treatments. **d** ChIP-qPCR assays validated the occupancy of Flag-tagged PHF14 on region 1 of *SMAD7* gene locus in Calu3 and HCC827 cells with indicated treatments. **e**, **f** qPCR and WB analysis showed the mRNA and protein levels of SMAD7 in Calu3 and HCC827 cells with indicated treatments. **g** BSP validated the DNA methylation level of region 1 of *SMAD7* gene locus in Calu3 and HCC827 cells with indicated treatments. **h**, **i** qPCR and WB analysis showed the mRNA and protein levels of SMAD7 in Calu3 and HCC827 cells with indicated treatments. V or shV represents control vectors for overexpression or shRNA knockdown, respectively. For aforementioned WB, qPCR, ChIP-qPCR and BSP assays, cells were treated with TGF-β1 at final concentration of 5 ng/mL for 48 h before the indicated assays were performed (**b**–**i**). Error bars represent means ± SD derived from three independent experiments. Two-way ANOVA multiple comparison analysis was used for statistical analysis. **P < 0.01; *P < 0.05; ns, not significant, *P* > 0.05.

Next, we further investigated the effect of PHF14 and DNMT3B interaction on DNA methylation of *SMAD7*. Immunofluorescent staining revealed that either endogenous PHF14 or ectopically overexpressed Flag-tagged PHF14 were nuclear localized protein (Fig. 3a), suggesting a possibility that PHF14 may directly regulate DNMT3B-catalyzed *SMAD7* DNA methylation. Notably, chromatin immunoprecipitation-quantitative polymerase chain reaction (ChIP-qPCR) assays showed that binding of DNMT3B to *SMAD7* DNA (regions of the 6 probes) could be abrogated by TGF-β treatment; however, the interaction was enhanced in different levels by PHF14 overexpression in cells with or without TGF-β treatment, but greatly diminished by knocking out PHF14 in LAD cells (Fig. 3b; Supplementary Fig. S4a, b). Simultaneously, the DNA methylation level of *SMAD7* was also elevated in PHF14-overexpressing cells treated with TGF-β, but could be repressed by loss of PHF14 expression or silencing DNMT3B (Figs. 2f, i, 3c; Supplementary Figs. S3b, c, S4c). Moreover, the binding of PHF14 on *SMAD7* DNA was barely altered by silencing DNMT3B, whereas the PHF14 overexpression-enhanced DNA methylation on *SMAD7* DNA could be reversed when DNMT3B expression was silenced (Fig. 3c, d; Supplementary Fig. S4c, d). Meanwhile, TGF-β treatment-induced SMAD7 elevation was suppressed by PHF14 overexpression, and silencing DNMT3B could abrogate the effect of PHF14 overexpression in LAD cells (Fig. 3e, f). In addition, treatment with 5-Aza-2-deoxycytidine (Decitabine), an inhibitor of DNA methyltransferases, showed a similar effect to that caused by DNMT3B depletion on *SMAD7* DNA methylation and expression level in LAD cells (Fig. 3g–i; Supplementary Fig. S4e), together suggesting that PHF14 plays a crucial role in DNMT3B recruitment to *SMAD7* DNA, and is required for *SMAD7* DNA methylation and transcriptional suppression.

### PHF14 serves as a CG-rich motif reader

**Fig. 4:**
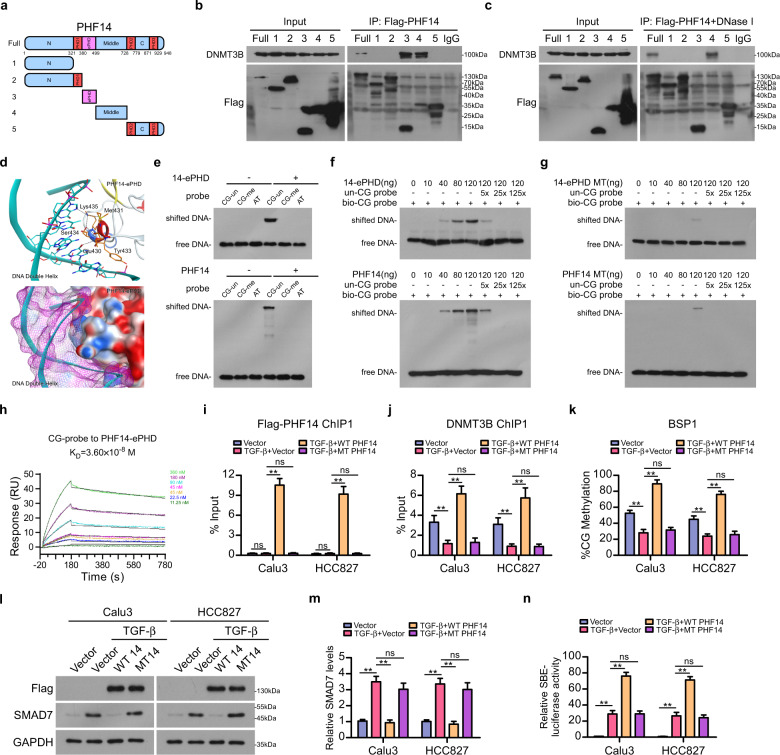
PHF14 serves as a CG-rich motif reader to recruit DNMT3B on *SMAD7* gene. **a** Schematic diagram of the truncated PHF14 protein constructions. Interactions between DNMT3B and various Flag-tagged truncated PHF14 constructions were evaluated by co-IP assays in 293FT cells without (**b**) or with DNase I treatment (**c**). **d** Wall-eye stereo view of the predicted interaction between the α-helix of PHF14 ePHD domain (right) and the major groove of DNA helix (left), in which key residues are shown as sticks, hydrogen bonds are shown as black dashes (upper panel), and electrostatic surface view of PHF14 ePHD domain bound to DNA helix (lower panel) was also shown. **e** Interactions between PHF14 ePHD domain or full-length PHF14 with unmethylated/methylated CG-rich oligonucleotides probes or AT-rich oligonucleotides probe were evaluated by EMSA using purified proteins. **f**, **g** Interactions between wild-type or mutated E430AK435A PHF14 ePHD domain or full-length PHF14 with unmethylated CG-rich oligonucleotides probes were evaluated by EMSA using purified proteins. Shifted DNA indicates protein-bound oligonucleotide probes, and free DNA indicates protein-free oligonucleotide probes. **h** SPR analysis measuring the affinity and kinetics of the interaction between PHF14 ePHD domain with CG-rich oligonucleotides probe. PHF14 ePHD domain was immobilized on a CM5 chip. **i** ChIP-qPCR assays validated the occupancy of Flag-tagged PHF14 on region 1 of *SMAD7* gene locus in Calu3 and HCC827 cells with indicated treatments. **j** ChIP-qPCR assays validated the recruitment of DNMT3B on region 1 of *SMAD7* gene locus in Calu3 and HCC827 cells with indicated treatments. **k** BSP validated the DNA methylation level of region 1 on *SMAD7* gene locus in Calu3 and HCC827 cells with indicated treatments. **l**, **m** WB and qPCR analysis showed the effect of expressing wild-type or mutated PHF14 on the protein and mRNA levels of SMAD7 in Calu3 and HCC827 cells with indicated treatments. **n** Measurement of SBE-luciferase activity showed relative TGF-β signaling activates in Calu3 and HCC827 cells with indicated treatments. For aforementioned WB, qPCR, ChIP-qPCR, BSP and SBE-luciferase activity assays, cells were treated with TGF-β1 at final concentration of 5 ng/mL for 48 h before the indicated assays were performed (**i**–**n**). Error bars represent means ± SD derived from three independent experiments. Two-way ANOVA multiple comparison analysis was used for statistical analysis. **P < 0.01; ns, not significant, *P* > 0.05.

To further understand the molecular basis upon which PHF14 recruits DNMT3B to *SMAD7* DNA, various Flag-tagged truncated proteins of PHF14 were constructed, as shown in the schematic diagram of the PHF14 protein domains (Fig. 4a). Subsequently, co-IP assays revealed that the ePHD and Middle domain of PHF14 interacted with DNMT3B, and interestingly, the interaction between the ePHD domain of PHF14 and DNMT3B was drastically abrogated by DNase I treatment of the whole cell lysate, suggesting that DNMT3B directly binds the Middle domain of PHF14, whereas the interaction between the ePHD domain of PHF14 and DNMT3B might rely on their recognition of and binding with chromosome (Fig. 4b, c; Supplementary Fig. S5a).

In light of the previous finding that the zinc finger PHD domains can serve as a reader of histones or DNAs, purified ePHD domain of or full-length recombinant human PHF14 protein was incubated with the MODified Histone Peptide Array, and unexpectedly, no specific histone peptide was found to interact with either the PHF14 ePHD domain or its full-length protein (Supplementary Fig. S5b). We thus further examined whether the ePHD domain of PHF14 interacts with DNA. To this end, HDOCK server program was employed to predict interaction between the PHF14 ePHD domain and various DNA probes^27^, and our results showed that an α-helix region of the PHF14 ePHD domain (429–435aa) was responsible for mediating its interaction with, and stretching into the major groove of DNA helix, which has been recognized as a docking platform for protein recognition (Fig. 4d). Moreover, the docking score between unmethylated CG-rich oligonucleotide and the PHF14 ePHD domain was lower than that between methylated CG-rich oligonucleotide or AT-rich oligonucleotide with the PHF14 ePHD domain, indicating a more intense putative binding between the ePHD domain of PHF14 with unmethylated CG-rich DNA element (Supplementary Table S3).

Next, electrophoretic mobility shift assay (EMSA) was performed and showed that purified ePHD domain of PHF14 increased the shifts of the biotinylated CG-rich unmethylated oligonucleotide probe, and that further addition of corresponding unmodified probes attenuated the shifted band of the biotin-labelled probes in a dose-dependent competitive manner, whereas the interaction between ePHD and methylated CG-rich oligonucleotide probe or AT-rich oligonucleotide probe was substantially weak (Fig. 4e, f; Supplementary Fig. S5c, d). Furthermore, an analogous result was obtained from the incubation of the purified full-length PHF14 protein with above mentioned three types of oligonucleotide probes (Fig. 4f; Supplementary Fig. S5c, d). In addition, the HDOCK server-predicted interaction intensity suggested that amino acid mutations of the PHF14 ePHD domain in its α-helix DNA-binding region (E430AK435A) abrogated the binding between the PHF14 ePHD domain and CG-rich DNA element (Supplementary Table S3). Consistently, transduction of PHF14 ePHD- and full-length PHF14-expressing plasmids, respectively, containing E430A and K435A mutations also revealed that the interaction between PHF14 ePHD or full-length PHF14 with methylated CG-rich probe could be drastically vanished by the α-helix mutations (Fig. 4g). Moreover, the binding constants of PHF14 ePHD domain with CG-rich or AT-rich oligonucleotide probes, respectively, have been measured by the surface plasmon resonance (SPR) assay, which illustrated that the equilibrium dissociation constant (*K*_D_) of unmethylated CG-rich probe with ePHD domain of PHF14 was 3.60 × 10^−8^ M; in contrast, the *K*_D_ value of AT-rich probe with PHF14 ePHD domain or CG-rich probe with mutated ePHD domain was 2.30 × 10^−7^ M and 2.75 × 10^−7^ M (Fig. 4h; Supplementary Fig. S5e), suggesting that the ePHD domain of PHF14 specifically binds with CG-rich DNA motif in vivo.

Furthermore, the binding of PHF14 on *SMAD7* gene and the recruitment of DNMT3B on selected regions by PHF14 were markedly diminished by E430AK435A mutations of PHF14, as compared with those by WT PHF14 (Fig. 4i, j; Supplementary Fig. S5f, g). Meanwhile, DNA methylation level and the expression level of *SMAD7* were not altered by the E430AK435A PHF14 mutant as compared to the vector-control in cells with TGF-β treatment, and the pro-TGF-β activation effect of PHF14 was largely reversed by E430AK435A mutations (Fig. 4k–n; Supplementary Fig. S5h). These results strongly suggest that the ePHD domain of PHF14 is responsible for the binding of PHF14 with CG-rich motifs and unveils a heterotrimer complex constituted by DNMT3B, PHF14 and DNA, which supports the notion that PHF14 facilitates the DNMT3B-catalyzed de novo DNA methylation on *SMAD7* locus.

### PHF14 promotes TGF-β-induced LAD progression and metastasis

**Fig. 5:**
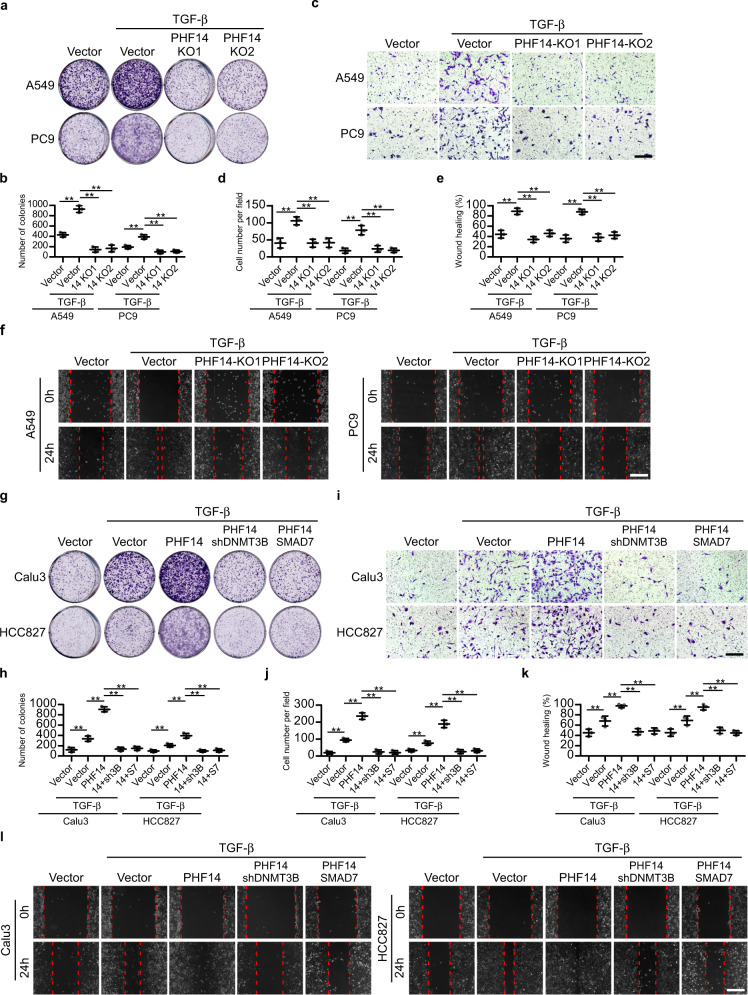
PHF14 promotes TGF-β-induced colony formation, invasion and migration of LAD cells in vitro. **a**, **b** Representative images and quantification of colony formation assays on A549 and PC9 cells with indicated treatments. **c**, **d** Representative images and quantification of invading cells in five random fields of matrigel-coated transwell assays performed on A549 and PC9 cells with indicated treatments. **e**, **f** Representative micrographs and quantification of wound healing assays performed on A549 and PC9 cells with indicated treatments. Images were photographed 24 h after wounds were scratched using pipette tips. **g**, **h** Representative images and quantification of colony formation assays performed on Calu3 and HCC827 cells with indicated treatments. **i**, **j** Representative images and quantification of invading cells in five random fields of matrigel-coated transwell assays performed using Calu3 and HCC827 cells with indicated treatments. **k**, **l** Representative micrographs and quantification of wound healing assays performed on Calu3 and HCC827 cells with indicated treatments. Images were photographed 24 h after wounds were scratched using pipette tips. PHF14, SMAD7 or shRNA of DNMT3B were ectopically stably transduced into indicated cells using lentiviral vectors. Prior to the cell functional assays, cells were pre-treated with TGF-β1 at final concentration of 5 ng/mL for 24 h, and this treatment was sustained throughout the assays (**a**–**l**). Scale bar: 100 μm (**c**, **i**), 300 μm (**f**, **l**). Error bars represent means ± SD derived from three independent experiments. Two-way ANOVA multiple comparison analysis was used for statistical analysis. **P < 0.01.

To determine the biological function of PHF14 in TGF-β-mediated LAD malignancies, the effects of PHF14 on in vitro cultured LAD cells were tested. As shown in Fig. 5a–f, our results showed that while TGF-β greatly enhanced colony formation, invasion and migration abilities of LAD cells, deletion of PHF14 in A549 and PC9 cells markedly impaired the TGF-β1-promoted malignant phenotype of the LAD cells as compared to vector controls (Fig. 5a–f). By contrast, overexpressing PHF14 in Calu3 and HCC827 LAD cells significantly enhanced the cell colony formation, invasion and migration abilities of the tested cells treated with TGF-β1, whereas silencing DNMT3B or overexpressing SMAD7 in PHF14-overexpressed cells potently reversed the phenotype synergistically enhanced by TGF-β1 and PHF14 co-expression (Fig. 5g–l), suggesting that the tumor promoting role of PHF14 largely depends on DNMT3B and SMAD7.

**Fig. 6:**
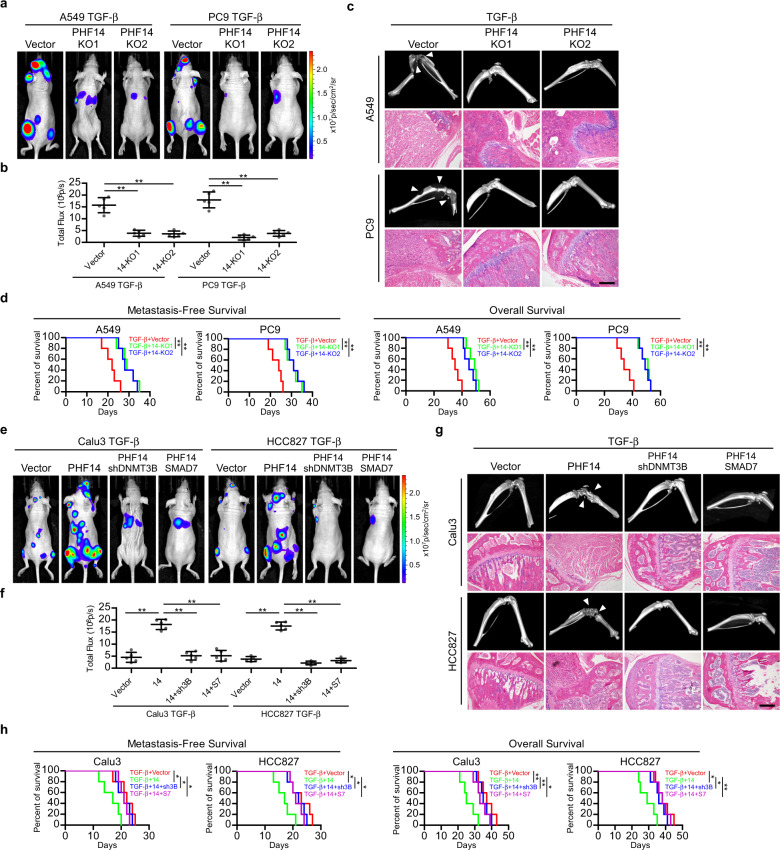
PHF14 promotes TGF-β-induced systemic metastases of LAD cells in vivo. **a**, **b** TGF-β1-overexpressing A549 and PC9 cells with PHF14 knocked out or corresponding vector-control cells labeled with reporting luciferase were injected via cardiac ventricle into nude mice (*n* = 5 per group). Representative bioluminescent images of systemic metastasis are shown (**a**), and quantitation of bioluminescent intensities were analyzed by ROI tools (**b**). **c** Metastatic bone lesions in mice intracardially injected with indicated cells were confirmed by micro-CT imaging and H&E staining. **d** Kaplan-Meier analysis (Log-rank test) of metastasis-free and overall survival of nude mice intracardially injected with indicated TGF-β1-overexpressing A549 and PC9 cells. **e**, **f** TGF-β1-overexpressing Calu3 and HCC827 cells labeled with luciferase reporter following indicated treatments were injected via cardiac ventricle into nude mice (*n* = 5 per group). Representative bioluminescent images of systemic metastasis are shown (**e**), and quantitation of bioluminescent intensities were analyzed by ROI tools (**f**). **g** Metastatic bone lesions in mice intracardially injected with the indicated cells were confirmed by micro-CT imaging and H&E staining. **h** Kaplan-Meier analysis (Log-rank test) of metastasis-free and overall survival of nude mice intracardially injected with indicated TGF-β1-overexpressing Calu3 and HCC827 cells. TGF-β1, PHF14, SMAD7 or shRNA of DNMT3B were ectopically stably expressed in indicated cell lines by using lentivirus. Scale bar: 100 μm. Error bars represent means ± SD derived from independent experiments. Two-way ANOVA multiple comparison analysis was used for statistical analysis. **P < 0.01; *P < 0.05.

In consistence with the above observed effects of PHF14 in vitro, intracardial inoculation of LAD cells labeled with luciferase showed that loss of PHF14 drastically abrogated the abilities of TGF-β1-overexpressing A549 and PC9 cells to form distant metastases, as evidenced by weaker bioluminescent signals in mice with PHF14 knockout cells than that of mice with vector-control cells (Fig. 6a, b). Indeed, micro-computed tomography (micro-CT) imaging and histological staining revealed markedly diminished TGF-β-driven cancerous lesions and tissue invasion/disruption in the lower-limb bones of nude mice intracardially injected with TGF-β1-overexpressing and PHF14 knocked out LAD cells, whereas TGF-β1-overexpressing vector-control cells displayed a robust ability to spread to bone tissues (Fig. 6c). Consequently, nude mice intracardially inoculated with PHF14-deleted LAD cells exhibited significantly prolonged metastasis-free and overall survival as compared with those inoculated with vector-control cells (Fig. 6d). On the contrary, PHF14 markedly promoted TGF-β1-overexpressing Calu3 and HCC827 LAD cells to form systemic metastases, with enhanced multi-organ bioluminescent signals, prominent cancerous lesions, severe tissue invasion/disruption in the lower-limb bones, and shortened metastasis-free and overall survival of nude mice intracardially injected with TGF-β1 and PHF14 co-overexpressing LAD cells as compared to those inoculated with the vector-control cells (Fig. 6e–h). Notably, silencing DNMT3B or overexpressing SMAD7 robustly abrogated the ability of TGF-β1 and PHF14 co-overexpressing LAD cells to form systemic metastases and prolonged metastasis-free and overall survival of nude mice intracardially injected with these LAD cells (Fig. 6e–h). Taken together, our data clearly demonstrated a crucial and potent pro-metastatic role of PHF14 in TGF-β-induced LAD progression and metastasis in a manner dependent on the PHF14-DNMT3B-SMAD7 axis.

### Potential significance of SMAD7 and PHF14 as promising diagnostic and prognostic biomarkers for LAD progression

**Fig. 7:**
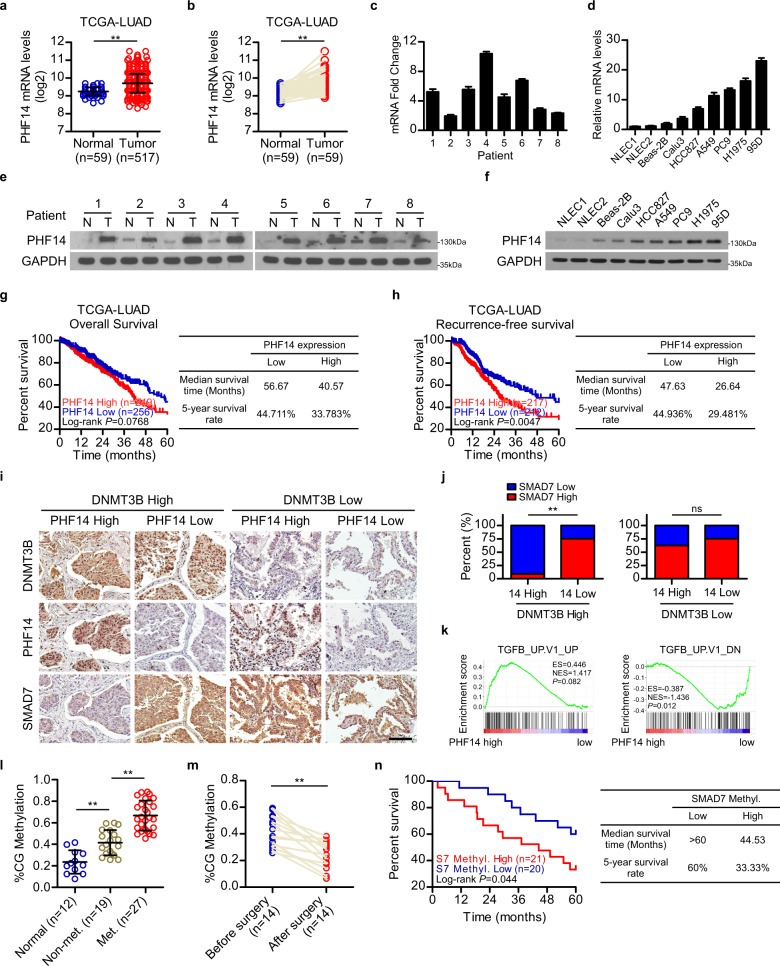
SMAD7 and PHF14 are diagnostic and prognostic biomarkers for LAD progression. **a**, **b** Analysis of *PHF14* expression in 517 cases of LAD tissue and 59 cases of adjacent normal lung tissue (**a**), and 59 pairs of paired human LAD tissue and adjacent normal lung tissue (**b**) using the TCGA LUAD datasets. **c**–**f** WB and qPCR analysis showed that the expression of PHF14 is upregulated in LAD tissues compared with adjacent non-cancerous lung tissues in 8 cases of freshly collected LAD specimens, and in LAD cell lines compared with primary NLEC. **g**, **h** Kaplan-Meier analysis (Log-rank test) of the 5-year overall survival (left panel) and recurrence-free survival (right panel) of LAD patients in the TCGA LUAD datasets, who were divided into low or high *PHF14* expression subgroups. **i**, **j** Representative IHC images show that PHF14 expression was inversely associated with the level of SMAD7 in LAD specimens with high DNMT3B expression, and the correlation between the expression levels of PHF14 and SMAD7 was not significant in those expressing low DNMT3B. Four representative cases are shown, scale bar: 50 μm. **k** GSEA analysis indicated that the *PHF14* level positively correlated with TGF-β-mediated transcription using the TCGA LUAD dataset. The defined “high” and “low” expression levels of *PHF14* were stratified by the median expression level. **l** Analysis of the *SMAD7* ctDNA methylation level from our collected cohort, including 12 cases normal serum samples, 19 cases serum samples from non-metastatic LAD patients and 27 cases serum samples from metastatic LAD patients. **m** Analysis of the *SMAD7* methylation level of ctDNA from 14 pairs serum samples of LAD patients before and after surgical resection of cancer. **n** Kaplan-Meier analysis (Log-rank test) of the 5-year overall survival of LAD patients in our collected LAD cohort, who were divided into two subgroups with low or high *SMAD7* ctDNA methylation level. Two-tailed unpaired Student’s *t*-test (**a**), two-tailed paired Student’s *t*-test (**b**, **m**), cross-tabulation with two-tailed Chi-square test (**j**) and two-way ANOVA multiple comparison analysis (**l**) were used for statistical analysis. **P < 0.01; ns, not significant, *P* > 0.05.

We next evaluate whether the expression of PHF14 and SMAD7 can indicate the state of clinical progression of LAD. In this context, we found that *PHF14* was significantly upregulated in LAD tissues collected in the TCGA LUAD datasets, as compared with the normal tissue specimens, which include 59 pairs of lung cancer/normal tissues and 517 cases of lung cancer vs 59 cases of unpaired normal lung tissue (Fig. 7a, b). PHF14 upregulation can also be observed in our LAD tissue cohort containing 8 cases of freshly collected LAD specimens paired with adjacent non-cancerous tissues and in a panel of LAD cell lines as compared to normal lung epithelial cells (NLECs) and an immortalized normal lung epithelial cell line Beas-2B (Fig. 7c–f). Moreover, using the TCGA LAD datasets, Kaplan-Meier analysis showed that LAD patients with high *PHF14* expression in their lung tumors displayed a significantly shorter median survival time of 40.57 months and lower 5-year survival rate of 33.783%, in contrast to the 56.67-month median survival time and 44.711% 5-year survival rate for those with low *PHF14* expression; in parallel, high *PHF14* levels correlates with shorter recurrence-free survival (26.64 months median survival time and 29.481% 5-year survival rate in high PHF14 expression subgroup vs 47.63 months median survival time and 44.936% 5-year survival rate in low PHF14 expression subgroup) (Fig. 7g, h). Importantly, the correlations between PHF14 and SMAD7 levels in DNMT3B-high and -low scenarios, respectively, were further examined in our collected patient tissue specimen cohort. Our results showed that PHF14 expression was inversely associated with the level of SMAD7 in tumor specimens with high DNMT3B expression, and by contrast, the correlation between the expression levels of PHF14 and SMAD7 was not significant in those expressing low DNMT3B (Fig. 7i, j). In addition, high *PHF14* level was found to positively correlate with TGF-β-mediated transcription in TCGA LUAD dataset (Fig. 7k), further supporting a close clinical relevance of the PHF14 and SMAD7 expression in LAD progression.

It is of particular note that when we examined *SMAD7* DNA methylation by detecting ctDNA obtained through liquid biopsy from LAD patients in our collected cohorts (Supplementary Table S4). We found that the *SMAD7*methylation levels of ctDNA in patients bearing metastatic cancer (*n* = 27) were significantly higher than those in patients only with primary LAD tumor (*n* = 19), and the ctDNA methylation levels in these two groups were markedly greater than those in the normal controls (*n* = 12) (Fig. 7l). In addition, *SMAD7* ctDNA methylation levels were significantly reduced in patients after LAD tumors were resected as compared with those in the same group of patients before tumor resection (*n* = 14) (Fig. 7m). Furthermore, there is a positive correlation between the *SMAD7* methylation levels of ctDNA and poor prognosis of LAD patients. Patients with higher *SMAD7* ctDNA methylation levels exhibited substantially shorter survival time as compared with those with lower *SMAD7* methylation level of ctDNA (Fig. 7n). Collectively, the above analyses suggest that the SMAD7 DNA methylation level in ctDNA correlates well with tumor burden and may have the potential to be applied to indicate tumor progression and prognosis of the patients.

## Discussion

It has been long noted that TGF-β signaling can be both tumor-suppressive at the initiation phase of tumorigenesis and pro-metastatic in later stages of cancer progression, known as the dual role of TGF-β during cancer development^28^. Of particular note, therefore, monotherapy targeting TGF-β ligand–receptor interaction usually reveals suboptimal and highly variable therapeutic effects, suggesting that the biological functions of TGF-β signaling and the outcome of TGF-β targeting cancer therapies may be largely context-dependent^29^. Also notably, accumulated evidence has indicated that TGF-β signaling potently drives SMAD-specific transcriptional program, and that orchestration of the biologically distinct roles of TGF-β signaling in cancer cells may in part rely on molecules that interact with SMADs and thereby modulate the functions of SMADs, which consequently alters the expression spectra of TGF-β target genes^30–35^. Nevertheless, how the functions of various SMADs are changed by their interactive molecules and consequently redirect the biological effects of TGF-β signaling remains incompletely understood.

SMAD7 functions as a critical antagonist of TGF-β signaling to prevent excessive activation of the pathway through promoting poly-ubiquitination-mediated proteasomal degradation of TGF-β receptor TGFBR1; in addition, SMAD7 also has been defined as a direct downstream transcriptional target of TGF-β signaling cascade, forming a negative feedback loop of TGF-β signaling^36,37^. Mounting evidence shows that upregulated *SMAD7* mRNA level, prolonged SMAD7 mRNA or protein stabilities, and dysregulated biological functions of SMAD7 in various types of cancers can be attributable to multiple transcription factors/co-factors^38,39^, non-coding RNAs^8,9,11,40,41^ and molecules involved in protein post-translational modifications^10,12,13,42–44^. In such a context, our present study identifies a PHF14/DNMT3B-mediated epigenetic mechanism via which *SMAD7* transcription is suppressed in LAD. Consistently, previous studies have found that the expression of DNMT3B and PHF14 can be elevated following TGF-β stimulation^45,46^. Moreover, our data unveil that the transcriptional status of *SMAD7* in LAD is jointly regulated by TGF-β signaling-mediated transcriptional activation and PHF14/DNMT3B-mediated transcriptional suppression. Only DNMT3B seems insufficient for de novo DNA methylation of *SMAD7*, and thus our results further elucidate an important role of PHF14 in this regulatory mechanism, underscoring that the upregulation of PHF14 expression is essential for aberrant activation of TGF-β signaling in our model. Taken together, our findings provide new insights into the molecular basis for TGF-β-driven cancer metastasis through spontaneous disruption of the negative feedback loop of TGF-β regulation. Whether such a molecular mechanism also exists and functions in other cancer types need to be further investigated.

As a component in the newly identified PHF14/DNMT3B complex, DNMT3B is known as one of the two de novo DNA methyltransferases responsible for generating 5-methylcytosine by transferring the methyl-group from S-adenosyl methionine to the fifth carbon of unmethylated cytosine^47^. Previous studies have found that DNMT3B could be upregulated by TGF-β signaling activation^45^, and that DNMT3B overexpression accelerates malignant transformation, promotes cancer progression and induces drug resistance in liver cancer, colon cancer and breast cancer, through enhancing DNA hypermethylation-mediated epigenetic suppression of tumor suppressor genes^18,19,48–50^. Interestingly, DNMT3A, a paralogue of DNMT3B, predominantly plays a tumor-suppressive role in cancers^51^. DNMT3A mutations are present in approximately 10%–20% hematological malignancies, with the R882 site being a unique mutation hotspot, conferring dominant-negative inhibition against the wild-type DNMT3A enzyme and poor prognosis^52^. Moreover, allelic loss of *DNMT3A* gene or loss of DNMT3A expression is frequently found in multiple human somatic tumor types; and depletion of *Dnmt3a* gene potently promotes progression of lung cancer with *Kras*^*G12D*^ mutant in a mouse model^26^. The functional discrepancies between DNMT3A and DNMT3B in stem cells have been revealed by several genome-wide studies and are thought to be attributed to distinctive preference of their genomic occupancies and specificities towards their target genes^53,54^. However, the mechanistic basis for the fundamental roles of DNMT3A and DNMT3B in tumorigeneses and cancer progression remains largely unknown, and it therefore becomes important to understand the underlying mechanism determining their occupancies on chromosomes and their distinct functions.

The amino acid sequence homology between DNMT3A and DNMT3B is approximately 70%, which is mainly contributed by their PWWP domains, the ADDz domains and the site-specific DNA-cytosine methylase domains. The rest residues, therefore, might contribute to DNMT3A’s and DNMT3B’s specific interactions with their various regulatory factors. Notably, decades of research on DNMTs have shown that the ADD and PWWP domains of DNMT3B are responsible for the recruitment of DNMT3B to histone H3 N-terminal tail^54–58^; and DNMT3B also interacts with histone methyltransferases SETDB1, EZH2 or transcription factors E2F6, PU.1 and NR6A1 to occupy specific regions on chromatins and silence the transcription of target genes^59–63^, suggesting that the recruitment of DNMT3B on chromosomes is sophisticatedly regulated by its binding partners in a context-dependent manner. In this current study, we found that DNMT3B is required to promote *SMAD7* DNA methylation and transcriptional suppression. In this context, zinc finger protein PHF14 is identified as a binding partner of DNMT3B, which recruits DNMT3B to the CpG sites distributed in the *SMAD7* gene locus, leading to DNA methylation-mediated transcriptional suppression of *SMAD7* and consequent dysregulated activation of TGF-β signaling. These results further suggest that the process of DNMT3B-mediated de novo DNA methylation on specific gene locus may involve uncharted epigenetic factors needed to be further identified and understood.

Our newly identified binding partner of DNMT3B, PHF14, has been previously shown to promote tumorigenesis and cancer progression by regulating cell mitosis, epithelial-mesenchymal transition, and Wnt and Akt signaling in multiple cancer types^64–68^. Mechanistically, PHF14 epigenetically suppresses the transcription of cell cycle inhibitors in cancers, including *p14*^*ARF*^, *p15*^*INK4b*^ and *p16*^*INK4a*^ genes^69^. Here, we find that PHF14 reads the CG-rich elements of DNA through its ePHD domain, and recruits DNMT3B to the *SMAD7* gene locus via its Middle domain, elucidating that PHF14 functions as a molecular scaffold linking DNMT3B and the CG-rich regions on *SMAD7* gene locus to restrain the transcription of *SMAD7* by enhancing its DNA hypermethylation. Recently, the PHD1-ZnK-PHD2 cassette of zebrafish PHF14 (corresponds to the PHD1-ePHD domain of human PHF14) has been found to be a reader for unmodified N-tail of histone H3^70^, implicating that the function of PHF14 may be complex and largely context-dependent, and needs to be further investigated.

In summary, our findings provide an insight into the mechanistic basis for aberrant activation of TGF-β signaling and its consequent induction of LAD metastasis, and unveil a novel PHF14/DNMT3B complex involved in the epigenetic suppression of *SMAD7*, indicating that PHF14 plays a pivotal role in determining the biological effects of TGF-β. These results also suggest a notable significance of PHF14 and SMAD7 in the diagnosis and prognostic evaluation LAD. Furthermore, it is of great interest to note that our current study also revealed the feasibility of employing liquid biopsy, a promising noninvasive diagnostic approach in clinical oncology^20,71,72^, to develop practically applicable tests for *SMAD7* ctDNA methylation as a clinically useful marker for LAD progression and prognosis. Moreover, it warrants future efforts to further evaluate whether inhibiting DNA methylation and abrogating the interaction among PHF14, DNMT3B and DNA can be a potential strategy for targeting TGF-β-driven LAD progression and metastasis.

## Materials and Methods

### Cell culture

LAD cell lines, including A549, PC9, Calu-3, HCC827, H1975, 95D and non-cancerous HEK293FT cells were purchased from the American Type Culture Collection (ATCC; Manassas, VA, USA) and maintained in Dulbecco’s modified Eagle’s medium (DMEM for LAD cell lines and HEK293FT cells; GIBCO, Carlsbad, CA, USA), supplemented with 10% fetal bovine serum (FBS; Corning, Tewksbury, MA, USA) and 1% penicillin/streptomycin (penicillin 100 U/mL and streptomycin 10 μg/mL) (GIBCO). The Beas-2B immortalized human bronchial epithelial cell line obtained from ATCC was cultured in Bronchial Epithelial Cell Growth Medium (BEGM) as instructed by the supplier (Lonza, Walkersville, MD, USA). All cell lines were authenticated by short tandem repeat DNA profiling at the Forensic Medicine Department of Sun Yat-Sen University Zhongshan School of Medicine (Guangzhou, China), and were verified to be mycoplasma-free.

### Plasmids, virus production and transfection

FLAG tagged-PHF14 (NP_055475), SMAD7 (NP_005895) and truncated mature human TGFB1 (NP_000651, aa. Ala270–Ser390) were separately cloned into the pSin-EF2-puro or pSin-EF2-neo retroviral vector (Addgene, Cambridge, MA, USA). FLAG-tagged or His-tagged full-length or truncated PHF14, HA-DNMT3A and HA-DNMT3B were separately cloned into the pCDNA3.4 TOPO vector (Invitrogen, Carlsbad, CA, USA). For depletion of PHF14, human gRNA sequences (PHF14-gRNA1: GCTGACTTGTTCCTCTGTAG; PHF14-gRNA2: GAAGTGGTCGGTTTCGTCGA) were cloned into the pLX330 vector (Genewiz, South Plainfield, NJ, USA). For silencing *SMAD7*, *DNMT3A* or *DNMT3B*, human shRNA sequences (SMAD7-shRNA1: GCTTTCAGATTCCCAACTTCT; SMAD7-shRNA2: GGTTTCTCCATCAAGGCTTTC; DNMT3A-shRNA: GCGTCACACAGAAGCATATCC; DNMT3B-shRNA: GCCCATTTGACTTGGTGATTG) against each of them were respectively cloned into the pSuper-retro-neo or pSuper-retro-puro vector (OligoEngine, Seattle, WA, USA). 3× SBE motifs (5′-GTCTAGAC-3′) were cloned upstream to the luciferase gene in a pGL3-Enhancer vector (Promega, Madison, WI, USA). For construction of cell lines knocked out for PHF14, pLX330-PHF14-gRNA plasmids were transfected by using Lipofectamine 3000 reagent (Invitrogen). The cells were subsequently dissociated with trypsin and re-suspended in DMEM containing 2% FBS. Then the cells were transferred into the 5 mL polypropylene round-bottom tubes (CORNING, NY, USA) on ice before being seeded into the 96-well plates by using flow cytometer (BD Influx) through GFP. For construction of stable cell lines overexpressing TGF-β1, PHF14 or SMAD7, and stable cell lines knocked down for SMAD7, DNMT3A or DNMT3B, plasmid pSin-TGFB1, pSin-PHF14 or pSin-SMAD7, respectively, was co-transfected with the pMD2G and psPAX2 packaging plasmids; and plasmid carrying specific shRNA for SMAD7, DNMT3A or DNMT3B, respectively, was co-transfected with the PIK packaging plasmid into 293FT cells using a standard calcium phosphate transfection method. Subsequently, cell culture supernatants of 293FT cells were collected to infect LAD cells for 24 h in the presence of polybrene (8 μg/mL), followed by selection with puromycin (1 μg/mL) or G418 (200 μg/mL) for 10–14 days. Transfection of plasmids or RNA oligonucleotides were performed using Lipofectamine 3000 reagent (Invitrogen) for luciferase reporter assays and molecular assays.

### Antibodies

Primary antibodies used for WB analyses were: Anti-SMAD3 (phospho S423 + S425) antibody (Abcam, ab52903, 1:2000), anti-SMAD3 antibody (Abcam, ab28379, 1:800), anti-MADH7/SMAD7 antibody (Abcam, ab216428, 1:1000), anti-PHF14 antibody (Proteintech, 24787-1-AP, 1:800), anti-DNMT3A antibody (Cell Signaling, 3598 S, 1:800), anti-DNMT3B antibody (Abcam, ab13604, 1:500), anti-FLAG antibody (Sigma-Aldrich, F1804, 1:2000), anti-HA antibody (Proteintech, 51064-2-AP, 1:2000), and Normal Mouse IgG for immunoprecipitation (Sigma-Aldrich, 12–371). Blotted membranes were stripped and re-blotted with anti-GAPDH antibody (Proteintech, 60004-1-Ig, 1:10000) as a loading control.

### RNA extraction and quantitative reverse transcription-PCR (qRT-PCR)

Total RNA from cultured cells or surgically resected fresh LAD tissues was isolated using TRIzol (Invitrogen) according to the manufacturer’s instruction. The first-strand cDNA was generated by reverse transcription using MMLV transcriptase (Promega) and random primers. Real-time qRT-PCR was performed on a CFX96 real-time PCR detection system (Bio-Rad, Richmond, CA, USA). Gene expression was assessed based on the threshold cycle (Ct), and relative expression levels were calculated as 2^–[(Ct of mRNA) – (Ct of GAPDH)]^ after normalization to GAPDH expression.

### SBE-luciferase assay

The TGF-β signaling activity was measured by dual-luciferase reporter assay (Promega) using TGF-β signaling activity reporter, which was constructed by cloning the 3× SBE motifs (CAGACAGACAGA) into pGL3-Basic plasmid as a promoter of Firefly Luciferase gene, and the Renilla Luciferase reporter (TK plasmid) used as an internal control. Relative luciferase activity (Firefly Luciferase/Renilla Luciferase) for each treatment was calculated as the TGF-β signaling activity.

### Immunoprecipitation and protein purification

Lysates were prepared from 3 × 10^7^ 293FT cells transfected with Flag-tagged full-length or truncated PHF14 or HA-tagged DNMT3B in an NP-40-containing lysis buffer supplemented with protease inhibitor cocktail (Roche), and then immunoprecipitated with FLAG or HA affinity agarose (Sigma-Aldrich) overnight at 4 °C. Beads containing affinity-bound proteins were washed six times with immunoprecipitation wash buffer (150 mM NaCl, 10 mM HEPES, pH 7.4, 0.1% NP-40), followed by elution with 0.1 M glycine (pH 3.0) twice. The eluted proteins were denatured and separated on SDS-polyacrylamide gels and stained with Coomassie blue; the indicated bands were subjected to MS analysis (Applied Protein Technology, Shanghai, China). Purification of recombinant His-tagged full-length or truncated PHF14 proteins was performed using BeaverBeads His-tag Protein Purification Kit (70501-K10, Beaver Biomedical Engineering Co., Suzhou, China).

### Immunofluorescence assay

A549 and 293FT cells were grown on coverslips and transfected with the indicated plasmid. At 24 h after transfection, cells were washed once with PBS and fixed in 4% paraformaldehyde in PBS. Subsequently, cells were permeabilized with 0.2% Triton X-100 and treated for 30 min at room temperature with 10% BSA in PBS, followed by incubation with primary antibody for 1 h. Primary anti-PHF14 antibody (Proteintech, 24787-1-AP) was used. The secondary antibody was goat anti-rabbit IgG (H + L) conjugated with Alexa Fluor 594 (Thermo Fisher Scientific) in 1% BSA at room temperature for 20–90 min. The cells were rinsed 2–3 times with PBS (pH7.4) (5 min/wash) and then mounted with Gold Antifade Mountant with DAPI (Thermo Fisher Scientific). The coverslips were washed extensively and fixed on slides, and representative images were obtained with a LSM810 confocal microscope using ZEN 2012 software version 8.1 (Carl Zeiss, Oberkochen, Germany).

### Bisulfite genomic sequencing

Genomic DNA from LAD cells, and clinical specimens’ ctDNA were bisulfite-modified with the Epitect Bisulfite Kit (Qiagen). Bisulfite-treated DNA was amplified with BSP primers designed in The Li Lab website (www.urogene.org) (BSP1 forward primer: 5′- AGTAAAGTAGTTTGAAGTGTGGTTTGTT -3′, reverse primer: 5′- CTATTAATACACAAAATATTCCCC -3′; BSP2 forward primer: 5′- GGGATATATTGGTTTTTTTTAAAGTAG -3′, reverse primer: 5′- AAACTTAATCCTTCTAACTAACTCC -3′; BSP3 forward primer: 5′- TTTGAAAATTTAATTTTGGGGAGTA -3′, reverse primer: 5′- TTTAACAATACTTAAAAAACTCAAAAC -3′; BSP4 forward primer: 5′- AAAATTTAGTTATAATAGAAAAATTTATTA -3′, reverse primer: 5′- AAATTAAAAATAAAACCTCTAAAAC -3′). PCR products were cloned using the pGEM-T Easy Vector System (Promega). Plasmids prepared from ampicillin-resistant bacteria clones were purified and sequenced.

### Prediction of PHF14–DNA interaction

The structure of human PHF14 ePHD was generated using MOE software version 2014.09 (Chemical Computing Group, Montreal, Canada) by modifying non-conserved amino acids in zebrafish PHF14 PZP structure downloaded from the Protein Data Bank (accession code 7D86). In addition, the structures of 3 types of DNA probes were also generated by the MOE software. Subsequently, both the structures of PHF14 ePHD and DNA probes were uploaded to the HDOCK server (http://hdock.phys.hust.edu.cn/) to predict the interaction models between PHF14 ePHD and DNA probes. The predicted models and interaction intensities were further analyzed.

### EMSA

EMSA was performed by using the LightShift Chemiluminescent EMSA kit purchased from Pierce Biotechnology (Rockford, IL, USA) according to the manufacturer’s standard protocol. Annealed dsDNA probes (CG-rich probe: sense, 5′- ACGGCTCGTCGCTACGGCTCGTCGCT -3′, antisense, 5′- AGCGACGAGCCGTAGCGACGAGCCGT -3′; AT-rich probe: sense, 5′- CATTAGATGATAGCATTAGATGATAG -3′, antisense, 5′- CTATCATCTAATGCTATCATCTAATG -3′, in which the positions of methyl-C are underlined) were incubated with diverse purified PHF14 proteins. Samples were then electrophoresed in a 0.5× TBE native polyacrylamide gel and the binding reactions were transferred to nylon membrane. Finally, the biotin-labeled DNAs were detected by chemiluminescence.

### SPR kinetic analysis

SPR kinetic analysis was performed with a method suggested by the instruction manual using HBS-P running buffer (10 mM HEPES, pH 7.4, 150 mM NaCl, 0.05% (v/v) surfactant P20) at 25 °C and employing a series S sensor chip CM5 on the Biacore T100 SPR instrument (Cytiva). The recombinant wild-type or E430AK435A mutated FLAG-tagged PHF14 ePHD protein was diluted in the running buffer and then immobilized to a density of 600–770 response units (RU). The chemosynthetic probes at different concentrations were sequentially injected on the chip surface for 180 s at a flow rate of 20 μL/min, with the dissociation phase monitored for up to 600 s. Individual sensor grams were referenced against injection onto an empty flow cell at equivalent concentrations. Data were fitted to a 1:1 Langmuir model using the BIAevaluate 4.0.1 analysis software.

### ChIP assay

ChIP assays were performed with a commercially available ChIP Assay Kit (Upstate Biotechnology, Lake Placid, NY, USA) by following the manufacturer’s instructions. In brief, cells (3 × 10^6^) cultured in 15-cm culture dishes were harvested for cross-linking and sheared by sonication. The resultant chromatin fractions were immunoprecipitated using anti-PHF14 (Proteintech, 24787-1-AP), anti-DNMT3B antibody (Abcam, ab13604), or negative control anti-IgG (Sigma-Aldrich) antibodies. After reversing the cross-links with NaCl and removing proteins with proteinase K, enriched DNA fragments were purified and isolated via phenol/chloroform extraction and ethanol precipitation. Real-time quantitative PCR of purified DNA fragments was performed with the indicated specific primers designed based on the putative PHF14 or DNMT3B binding sites in the *SMAD7* gene locus (ChIP1 forward primer: 5′- GGCGGACTTGATGAAGAT -3′, reverse primer: 5′- CAATTCGGACAACAAGAGT -3′; ChIP2 forward primer: 5′- GACACACTGGCTTCTTCC -3′, reverse primer: 5′- CTTGGTCCTTCTGGCTGA -3′; ChIP3 forward primer: 5′- GCCAAGGAGCGAACTATT -3′, reverse primer: 5′- CAGTTGCTGAGACTATCGT -3′; ChIP4 forward primer: 5′- ACCAAACTTCCAACAGAGG -3′, reverse primer: 5′- GTGTGTAGGCAAAGGCTT -3′).

### Colony formation assay

For colony formation assay, 1 × 10^3^ cells were plated in 6-well plates and cultured for 7 days, and colonies were stained with 1% crystal violet for 10 min after fixation with 10% formaldehyde for 5 min and counted.

### Wound-healing assay

Cells were seeded in 6-well plates and grown to 90% confluence, followed by serum starvation for 12 h. A linear wound was created in the confluent monolayer using a pipette tip, and wounds were observed and photographed at various time points. Wound size was measured randomly at 3 sites perpendicular to the wound.

### Animal experiments

Female BALB/c-nu mice (5–6 weeks of age, 18–20 g) were housed in specific pathogen-free facilities for animal studies. At least five nude mice per group were used to ensure the adequate power and each mouse with different weight was randomly allocated. For intracardiac injection, the indicated 5 × 10^5^ luciferase-expressing cells were resuspended in 0.1 mL PBS and inoculated into the left cardiac ventricle of nude mice. Metastases were monitored by bioluminescent imaging every 3 days. For bioluminescent imaging assay, 15 min prior to imaging, mice were injected intraperitoneally (i.p.) with 150 mg/kg luciferin. Following general anesthesia, images were taken and analyzed with Spectrum Living Image version 4.2 (Caliper Life Sciences, Waltham, MA, USA). At the experimental endpoint, nude mice were anesthetized and sacrificed, and lower limbs were resected, sectioned (5 μm in thickness) and histologically examined by H&E staining for the presence of metastatic lesions. All experimental procedures were approved by the Institutional Animal Care and Use Committee of Sun Yat-sen University.

### Immunohistochemistry (IHC) assay

IHC assays using anti-MADH7/SMAD7 antibody (Abcam, ab216428), anti-PHF14 (Proteintech, 24787-1-AP) and anti-DNMT3B antibody (Abcam, ab13604) were separately conducted on paraffin-embedded specimens of LAD patients. The immunostaining intensities of indicated proteins was evaluated and scored by two independent observers, scoring both the proportions of positive staining tumor cells and the staining intensities. Scores representing the proportion of positively stained tumor cells was graded as: 0 (no positive tumor cells), 1 (< 10%), 2 (10%–50%) and 3 (> 50%). The staining intensity was determined as: 0 (no staining), 1 (weak staining = light yellow), 2 (moderate staining = yellow brown) and 3 (strong staining = brown). The staining index (SI) was calculated as staining intensity × percentage of positive tumor cells, resulting in scores as 0, 1, 2, 3, 4, 6 and 9. Cut-off values for high- and low-expression of protein of interest were chosen based on a measurement of heterogeneity using the log-rank test with respect to overall survival.

### Clinical specimens and ethical approval

Two patient and control cohorts were employed in our current study. One cohort (i.e., the tumor and adjacent normal tissue cohort) includes LAD tissue specimens taken from 28 LAD patients, among which 8 adjacent-normal tissue specimens were also available (for Figs. 1f, g, 7c, e, i, j), and the second one was serum specimens derived from 12 healthy individuals and 46 LAD patients, respectively (for Fig. 7l–n). These two cohorts used in this study were obtained from and histopathologically diagnosed at Sun Yat-Sen Memorial Hospital. LAD tissues and paired adjacent non-cancerous lung specimens collected during surgery were frozen in liquid nitrogen and stored at –40 °C until further use. Adjacent non-cancerous specimens were obtained from a standard distance (3 cm) from the tumor margin in resected tissues of LAD patients. For the use of these clinical materials for research purposes, prior patients’ consents and approval from the Institutional Research Ethics Committee of Sun Yat-sen University were obtained. The study is compliant with all relevant ethical regulations involving human subjects.

### TCGA dataset analysis

RNAseq data for PHF14 expression of 59 pairs of LAD tissues and paired adjacent non-cancerous lung tissues, and 576 cases of LAD tissues, as well as their corresponding clinical information, were mined from TCGA lung cancer datasets (https://cancergenome.nih.gov/).

### Statistical analysis

All statistical analyses except sequencing data were performed using the PASW Statistics 18 version 18.0.0 (SPSS Inc., Chicago, IL, USA) software package and GraphPad Prism 8 version 8.3.0 (GraphPad Software, San Diego, CA, USA). Survival curves were analyzed by the Kaplan-Meier method and compared by the log-rank test. Comparisons between two groups were performed using Student’s *t*-test (two-tailed), while analyses comparing multiple treatments with a control group were performed using two-way ANOVA with post hoc Dunnett’s multiple comparisons test. All error bars represent means ± SD derived from three independent experiments. In all cases, *P* < 0.05 was considered to be statistically significant.

### Study approval

All experimental procedures and use of LAD donors’ samples were approved by the SYSU Institutional Animal Care and Use Committee, and Research Ethics Committee. Donors provided prior written informed consent.

## Supplementary information

Supplementary information

## Data Availability

All data generated or analyzed during this study are included in this article and supplementary information files. The TCGA Lung Adenocarcinoma (TCGA-LUAD) sequencing data used in this study are available in a public repository from the GDC Data Portal (https://portal.gdc.cancer.gov/).
