# Supplementary material for: PHF14 enhances DNA methylation of SMAD7 gene to promote TGF-β-driven lung adenocarcinoma metastasis
Source: Cell Discov. 2023 Apr 18;9:41. doi: 10.1038/s41421-023-00528-0 (PMC10113255; doi:10.1038/s41421-023-00528-0)
Supplement: Supplementary file 1 — Supplementary information [file 41421_2023_528_MOESM1_ESM.pdf]

Supplementary Information

PHF14 enhances DNA methylation of *SMAD7* gene to promote TGF-β-driven lung adenocarcinoma metastasis

Han Tian [1, 2, #], Chenying Liu [3, #], Jianchen Yu [1, #], Jian Han [2], Jianan Du [1], Shujun Liang [1], Wenting Wang [1], Qin Liu [1], Rong Lian [1], Ting Zhu [4], Shanshan Wu [5], Tianyu Tao [6], Yaokai Ye [1], Jingjing Zhao [7], Yi Yang [1], Xun Zhu [1], Junchao Cai [1], Jueheng Wu [1], Mengfeng Li [2, 1, *].

Supplementary Figs. S1–S5 and figure legends

Supplementary Tables S1–S4

Supplementary Figures

Supplementary Figure 1.

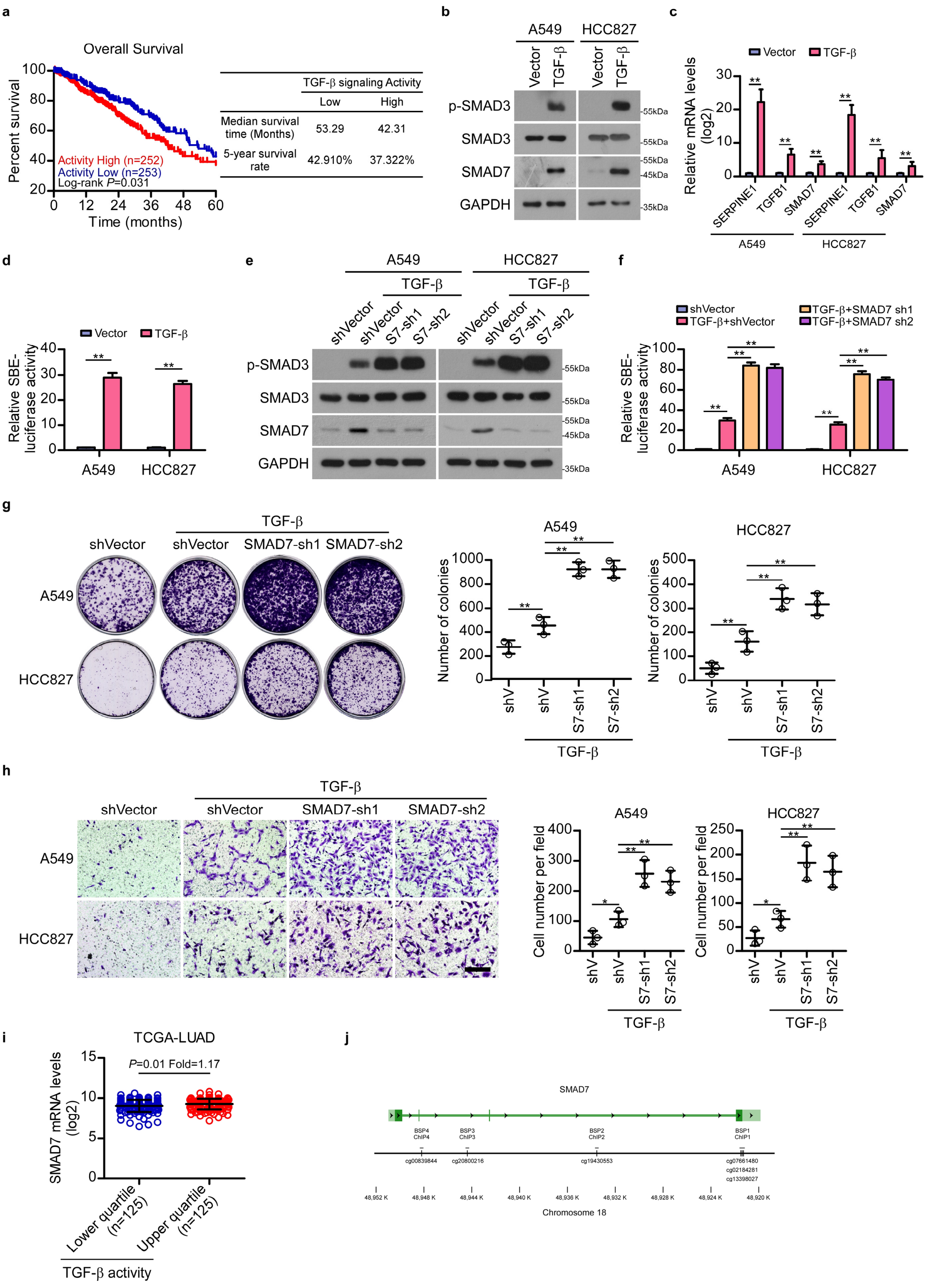

Supplementary Fig. S1: SMAD7 functions as an antagonist of TGF-β signaling in LAD.

a Kaplan-Meier analysis (Log-rank test) of the 5-year overall survival of LAD patients in the TCGA LUAD datasets, who were divided into low- or high-TGF-β signaling activity subgroups. **b** Western blotting (WB) analyses showed the protein levels of p-SMAD3, SMAD3 and SMAD7 in A549 and HCC827 cells treated with TGF-β1. **c** qPCR analyses showed the mRNA levels of *SERPINE1*, *TGFB1* and *SMAD7* in A549 and HCC827 cells treated with TGF-β1. **d** Measurement of SBE-luciferase activity revealed relative TGF-β signaling activates in A549 and HCC827 cells treated with TGF-β1. **e** WB analyses showed the effect of silencing SMAD7 on protein levels of p-SMAD3, SMAD3 in A549 and HCC827 cells. **f** Measurement of SBE-luciferase activity revealed relative TGF-β signaling activates in A549 and HCC827 cells with SMAD7 silenced. **g** Representative images and quantification of colony formation assays performed using A549 and HCC827 cells with indicated treatments. **h** Representative images and quantification of invading cells in five random fields of matrigel-coated transwell assays performed using A549 and HCC827 cells with indicated treatments. Scale bar: 100 μm. **i** Analysis of *SMAD7* mRNA levels in patients collected in the TCGA LUAD dataset, who were divided into low- or high-TGF-β signaling activity subgroups. **j** Schematic diagram of DNA methylation probes and corresponding regions of BSP and ChIP-qPCR on *SMAD7* gene locus. For aforementioned WB, qPCR and SBE-luciferase activity assays, cells were treated with TGF-β1 at final concentration of 5 ng/mL for 24 h before the indicated assays were performed (**b**–**f**), and for cell functional assays, cells were pre-treated with TGF-β1 at final concentration of 5 ng/mL for 24 h, and TGF-β1 (5 ng/mL) treatment was sustained during the assays (**g**–**h**). Error bars represent mean ± SD derived from three independent experiments. Two-tailed Student's *t*-test (**c**, **d** and **i**) and two-way ANOVA multiple comparison analysis (**f**–**h**) were used for

statistical analysis. $^{**}P < 0.01$; $^{*}P < 0.05$.

Supplementary Figure 2.

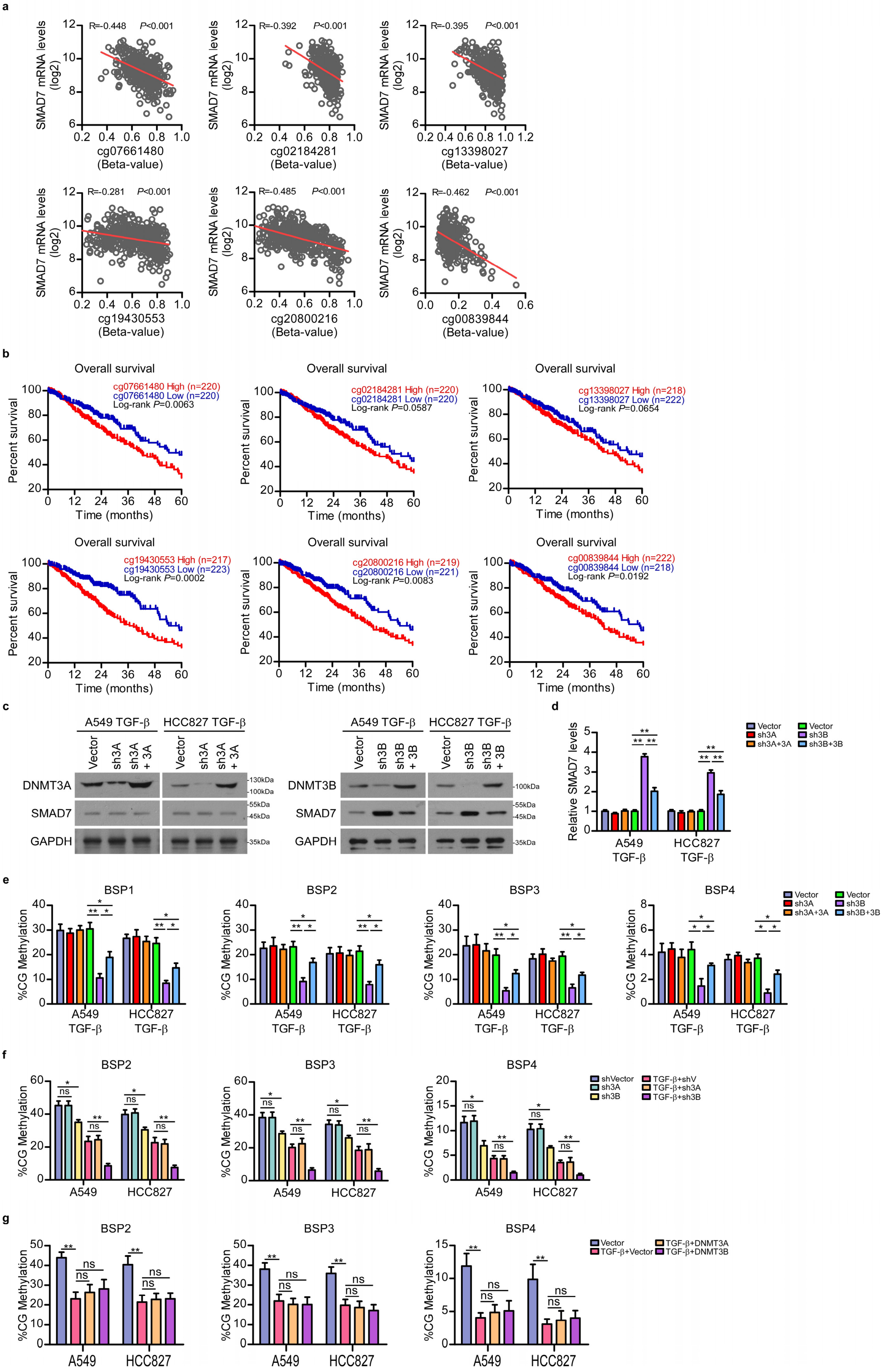

Supplementary Fig. S2: DNMT3B-catalyzed *SMAD7* DNA methylation

correlates with poor prognosis of LAD patients.

a Correlation between *SMAD7* mRNA levels and the beta-values (DNA methylation level) of the selected 6 methylation probes, respectively, in 460 cases of LAD tissues in the TCGA LUAD datasets (Spearman's correlation coefficient). **b** Kaplan-Meier analysis (Log-rank test) of the 5-year overall survival of LAD patients in the TCGA LUAD datasets, who were divided into low- or high-methylation level subgroups for each probe, respectively. **c** WB analyses showed the protein levels of DNMT3A, DNMT3B and SMAD7 in A549 and HCC827 cells with indicated treatments. **d** qPCR analysis showed the mRNA level of *SMAD7* in A549 and HCC827 cells with indicated treatments. **e**–**g** BSP validated the DNA methylation levels of regions 1, 2, 3 and 4 of *SMAD7* gene locus in A549 and HCC827 cells with indicated treatments. For aforementioned WB, qPCR and BSP assays, cells were treated with TGF-β1 at final concentration of 5 ng/mL for 48 h before the indicated assays were performed (**c**–**g**). Error bars represent mean ± SD. Two-way ANOVA multiple comparison analysis was used for statistical analysis (**d**–**g**). $^{**}P < 0.01$; $^{*}P < 0.05$; ns, not significant, $P > 0.05$.

Supplementary Figure 3.

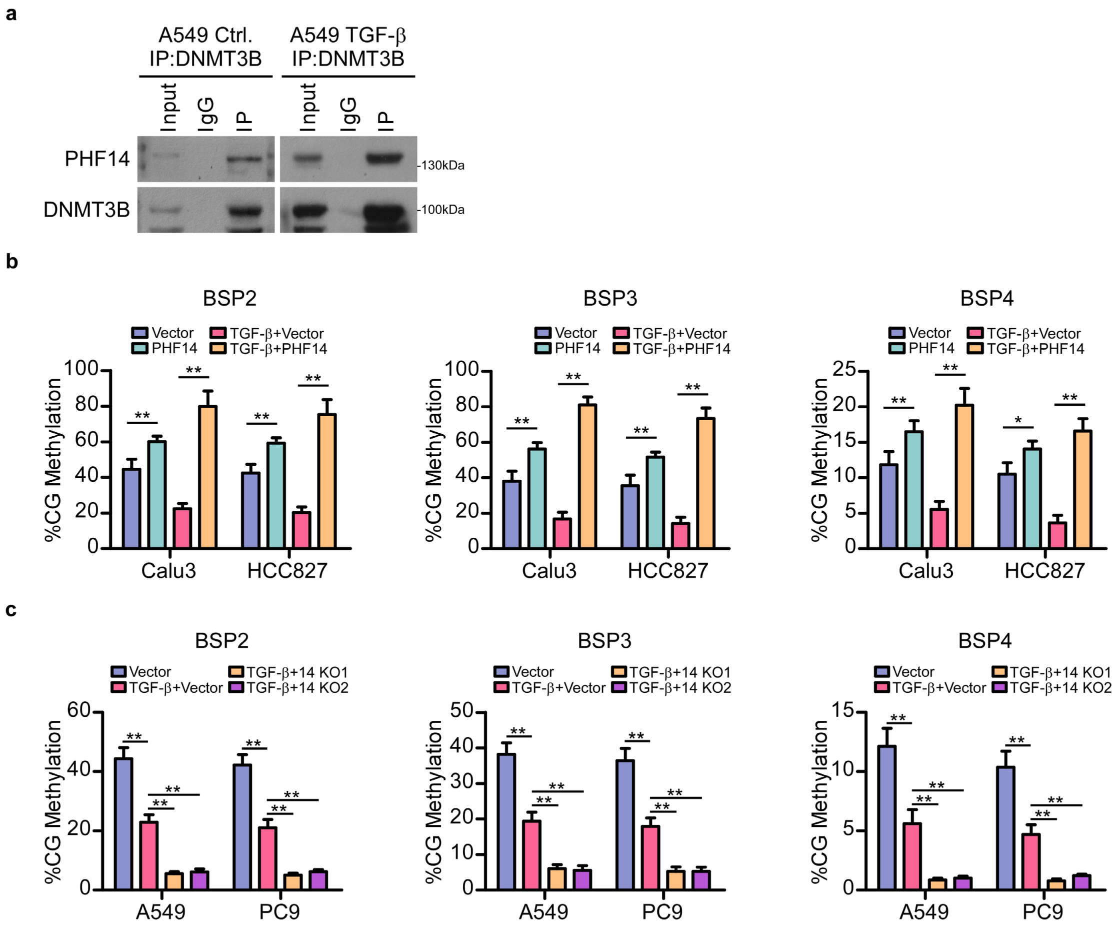

Supplementary Fig. S3: PHF14 is identified as a novel binding partner of DNMT3B.

a Immunoprecipitation assays of endogenous DNMT3B with PHF14 in A549 cells with or without TGF-β treatment to validate the interaction between DNMT3B and PHF14. **b** BSP validated the DNA methylation levels of regions 2, 3 and 4 of *SMAD7* gene locus in Calu3 and HCC827 cells with indicated treatments. **c** BSP validated the DNA methylation levels of regions 2, 3 and 4 of *SMAD7* gene locus in A549 and PC9 cells with indicated treatments. For aforementioned IP and BSP assays, cells were treated with TGF-β1 at final concentration of 5 ng/mL for 48 h before the indicated assays were performed (**a**–**c**). Error bars represent mean ± SD from three independent experiments. Two-way ANOVA multiple comparison

analysis was used for statistical analysis. **$P < 0.01$; *$P < 0.05$; ns, not significant, $P > 0.05$.

Supplementary Figure 4.

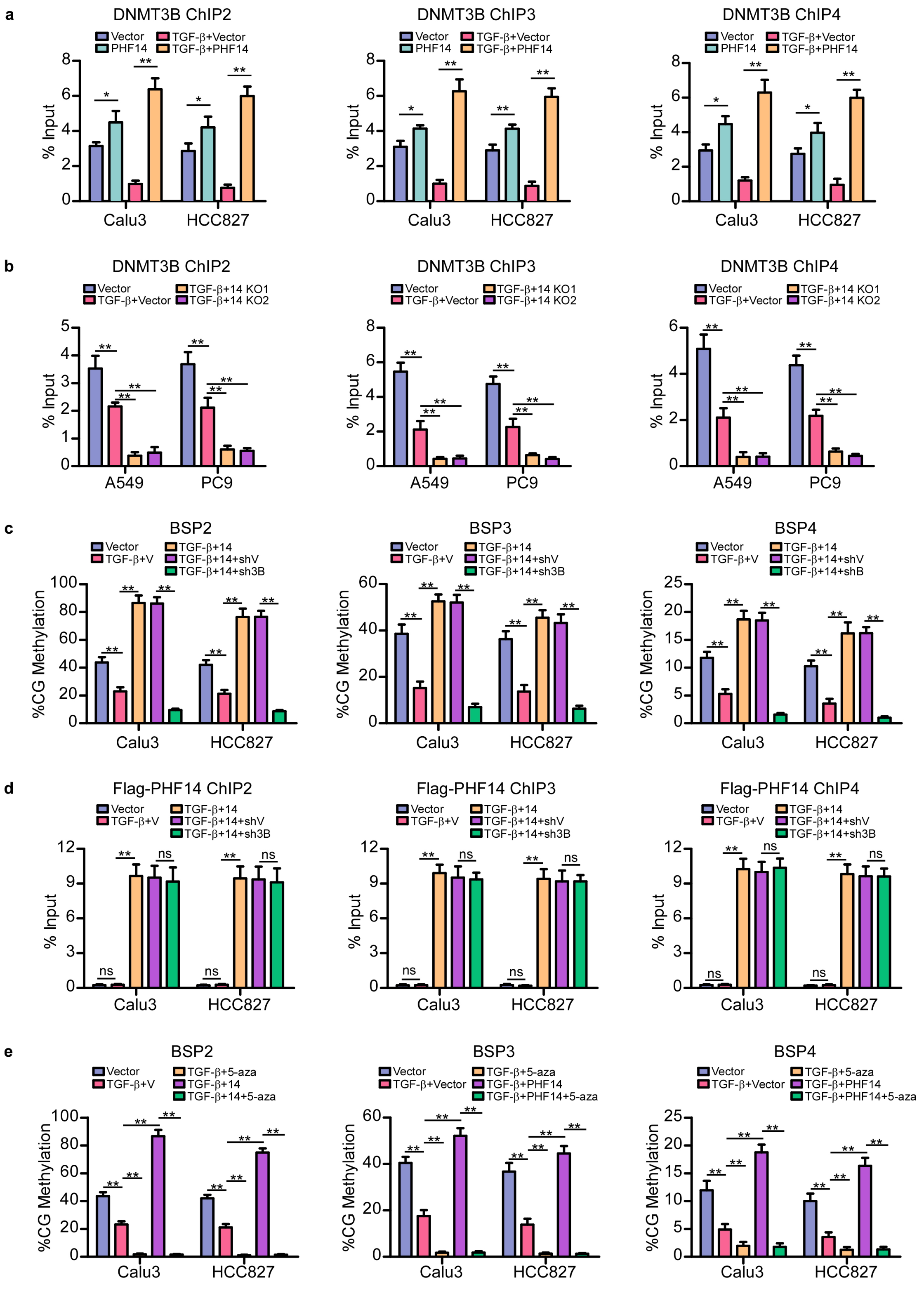

Supplementary Fig. S4: PHF14 facilities DNMT3B mediated DNA methylation of SMAD7.

a, b ChIP-qPCR assays validated the effect of overexpressing PHF14 **(a)** or depleting

PHF14 **(b)** on the occupancy of DNMT3B on regions 2, 3 and 4 of *SMAD7* gene locus in indicated cells. **c** BSP validated the DNA methylation levels of regions 2, 3 and 4 of *SMAD7* gene locus in Calu3 and HCC827 cells with indicated treatments. **d** ChIP-qPCR assays validated the occupancy of Flag-tagged PHF14 on regions 2, 3 and 4 of *SMAD7* gene locus in Calu3 and HCC827 cells with indicated treatments. **e** BSP validated the DNA methylation levels of regions 2, 3 and 4 of *SMAD7* gene locus in Calu3 and HCC827 cells with indicated treatments. For aforementioned ChIP-qPCR and BSP assays, cells were treated with TGF-β1 at final concentration of 5 ng/mL for 48 h before the indicated assays were performed (**a**–**e**). Error bars represent mean ± SD derived from three independent experiments. Two-way ANOVA multiple comparison analysis was used for statistical analysis (**a**–**e**). $^{**}P < 0.01$; $^{*}P < 0.05$; ns, not significant, $P > 0.05$.

Supplementary Figure 5.

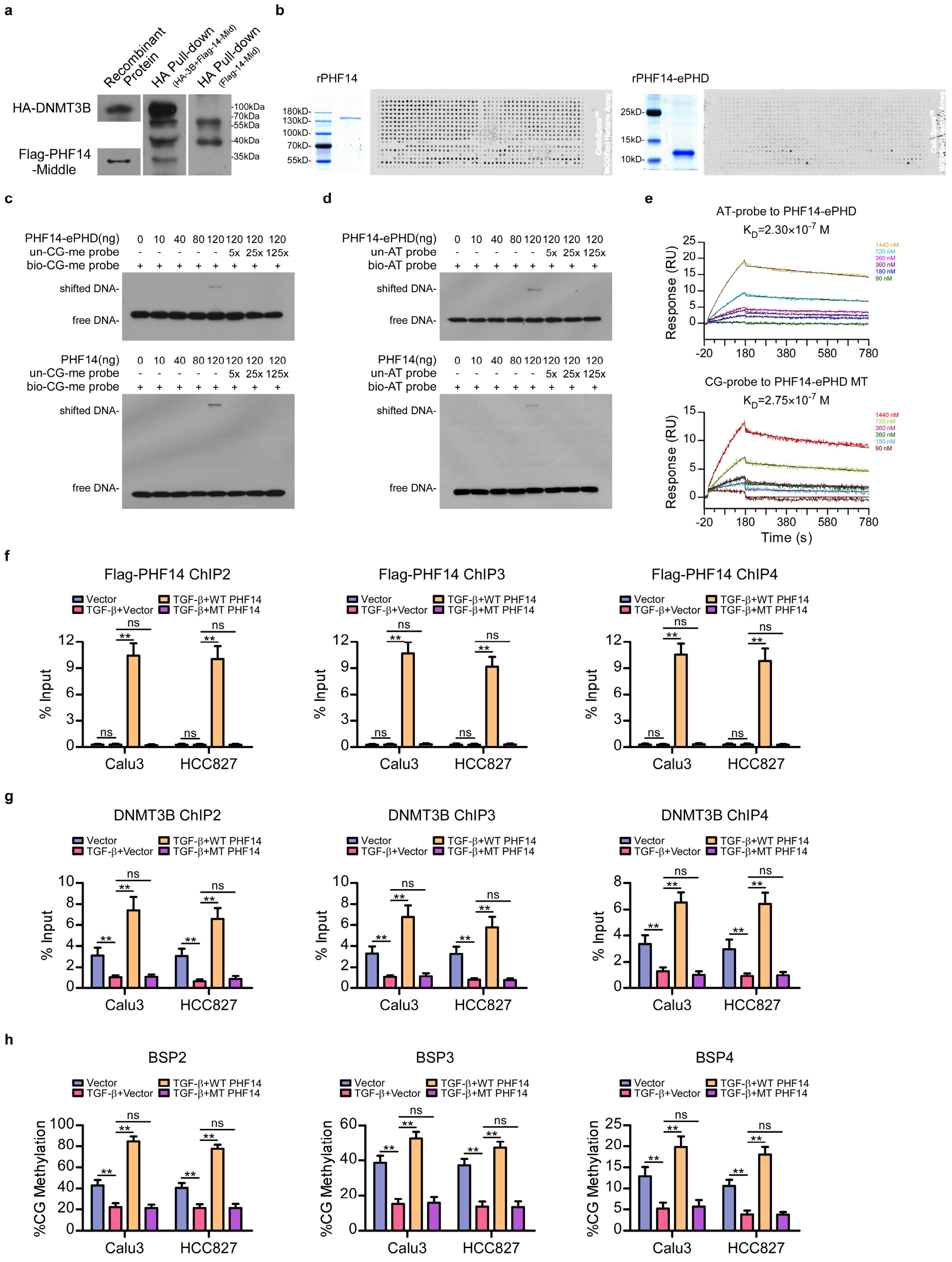

Supplementary Fig. S5: PHF14 serves as a CG-rich motif reader.

a Protein pull-down assay showed the interaction between purified HA-DNMT3B and Flag-PHF14-Middle domain *in vitro*. **b** Purified full-length human PHF14 protein or its ePHD domain did not interact with specific histone peptide on the MODified

histone peptide array. **c, d** Interactions between PHF14 ePHD domain or full-length PHF14 with methylated CG-rich (**c**) or AT-rich (**d**) oligonucleotides probe were evaluated by EMSA assays using purified proteins. Shifted DNA indicates protein-bound oligonucleotide probes, and free DNA indicates protein-free oligonucleotides probes. **e** SPR analysis measuring the affinity and kinetics of the interaction between PHF14 ePHD domain with AT-rich oligonucleotides probe and mutated PHF14 ePHD domain with CG-rich oligonucleotides probe. Wild-type or mutated E430A K435A PHF14 ePHD domain was immobilized on a CM5 chip. **f** ChIP-qPCR assays validated the occupancy of Flag-tagged PHF14 on regions 2, 3 and 4 of *SMAD7* gene locus in Calu3 and HCC827 cells with indicated treatments. **g** ChIP-qPCR assays validated the recruitment of DNMT3B on regions 2, 3 and 4 of *SMAD7* gene locus in Calu3 and HCC827 cells with indicated treatments. **h** BSP validated the DNA methylation levels of regions 2, 3 and 4 of *SMAD7* gene locus in Calu3 and HCC827 cells with indicated treatments. For aforementioned ChIP-qPCR and BSP assays, cells were treated with TGF-β1 at final concentration of 5 ng/mL for 48 h before the indicated assays were performed (**f**–**h**). Error bars represent mean ± SD derived from three independent experiments. Two-way ANOVA multiple comparison analysis was used for statistical analysis (**f**–**h**). $^{**}P < 0.01$; ns, not significant, $P > 0.05$.

Supplementary Table S1. 26 probes of SMAD7 in SMART

Probe	Location			Correlation (Gene-level)	
	Chromosome	Start	End	*R*	*p*
cg06203649	chr18	48921264	48921265	−0.320	< 0.001
cg13398027	chr18	48921470	48921471	−0.395	< 0.001
cg14283454	chr18	48923019	48923020	−0.132	0.005
cg19430553	chr18	48933564	48933565	−0.281	< 0.001
cg20800216	chr18	48944979	48944980	−0.485	< 0.001
cg21718735	chr18	48947203	48947204	−0.301	< 0.001
cg11645306	chr18	48947694	48947695	−0.357	< 0.001
cg00145587	chr18	48948874	48948875	−0.092	< 0.001
cg13967780	chr18	48950452	48950453	0.015	0.755
cg05119084	chr18	48950757	48950758	−0.028	0.553
cg15166561	chr18	48950776	48950777	−0.149	0.001
cg12651664	chr18	48950779	48950780	−0.055	0.237
cg21505940	chr18	48950803	48950804	−0.040	0.394
cg11023721	chr18	48951600	48951601	−0.025	0.595
cg00214056	chr18	48952014	48952015	−0.047	< 0.001
cg07661480	chr18	48921367	48921368	−0.448	< 0.001
cg02184281	chr18	48921427	48921428	−0.392	< 0.001
cg25986322	chr18	48921591	48921592	−0.217	< 0.001
cg11909137	chr18	48934832	48934833	−0.299	< 0.001
cg24375218	chr18	48935029	48935030	−0.303	< 0.001
cg00839844	chr18	48948274	48948275	−0.462	< 0.001
cg23699700	chr18	48949738	48949739	−0.013	0.779
cg19575813	chr18	48950567	48950568	0.042	0.373
cg05310557	chr18	48950880	48950881	−0.066	0.161
cg14751914	chr18	48951034	48951035	−0.067	0.151
cg15153018	chr18	48951442	48951443	−0.118	0.011

Supplementary Table S2. DNMT3B-interacting proteins identified by MS

#	Protein	Gene_Name	Unique_Peptide_Count	Molecular_Weight
1	Q9UBC3	DNM3B	26	95750
2	Q9Y6K1	DNM3A	24	101857
3	P26358	DNMT1	18	183165
4	O14744	ANM5	17	72683
5	P08107	HSP71	16	70051
6	P08238	HS90B	16	83263
7	Q15910	EZH2	16	85363
8	P45973	CBX5	15	22225
9	P07900	HS90A	14	84659
10	O94880	PHF14	13	100052
11	P04350	TBB4A	13	49585
12	Q9UJW3	DNMT3L	13	43583
13	O14654	IRS4	12	133766
14	P11142	HSP7C	12	70897
15	Q9BVA1	TBB2B	12	49953
16	O95071	UBR5	10	309349
17	Q9NVI7	ATD3A	10	71368
18	Q92616	GCN1L	9	292755
19	P10809	CH60	8	61054
20	P31689	DNJA1	8	44868
21	P34931	HS71L	8	70374
22	P38646	GRP75	8	73680
23	Q5W0B1	RN219	8	81116
24	Q93008	USP9X	8	292276
25	P12956	XRCC6	7	69842
26	P15880	RS2	7	31324
27	P19338	NUCL	7	76613
28	P60709	ACTB	7	41736
29	P61247	RS3A	7	29945
30	P62753	RS6	7	28680
31	P63261	ACTG	7	41792
32	P68363	TBA1B	7	50151

33	Q02978	M2OM	7	34061
34	O43175	SERA	6	56650
35	O60884	DNJA2	6	45745
36	P04843	RPN1	6	68569
37	P11586	C1TC	6	101558
38	P17066	HSP76	6	71027
39	P23396	RS3	6	26688
40	P26373	RL13	6	24261
41	Q16531	DDB1	6	126966
42	Q9BUF5	TBB6	6	49857
43	Q9Y4R8	TELO2	6	91746
44	O75190	DNJB6	5	36087
45	O95831	AIFM1	5	66900
46	P11021	GRP78	5	72332
47	P25705	ATPA	5	59750
48	P46781	RS9	5	22591
49	P62269	RS18	5	17718
50	Q02878	RL6	5	32728
51	Q92621	NU205	5	227919
52	Q96HS1	PGAM5	5	32004
53	Q9BQA1	MEP50	5	36724
54	Q9NVI1	FANCI	5	149322
55	Q9ULX6	AKP8L	5	71648
56	Q9UNF1	MAGD2	5	64953
57	A6NHL2	TBAL3	4	49908
58	O95816	BAG2	4	23772
59	P05141	ADT2	4	32852
60	P05388	RLA0	4	34273
61	P06493	CDK1	4	34095
62	P08195	4F2	4	67993
63	P0CG47	UBB	4	25761
64	P0CG48	UBC	4	77038
65	P13010	XRCC5	4	82704
66	P18077	RL35A	4	12538

67	P18124	RL7	4	29225
68	P18621	RL17	4	21397
69	P27348	1433T	4	27764
70	P30050	RL12	4	17818
71	P49368	TCPG	4	60533
72	P49411	EFTU	4	49541
73	P50402	EMD	4	28994
74	P50990	TCPQ	4	59620
75	P52272	HNRPM	4	77515
76	P53618	COPB	4	107141
77	P61978	HNRPK	4	50976
78	P61981	1433G	4	28302
79	P62258	1433E	4	29174
80	P62979	RS27A	4	17965
81	P62987	RL40	4	14728
82	P63244	GBLP	4	35076
83	Q08211	DHX9	4	140957
84	Q99615	DNJC7	4	56440
85	Q9BRS2	RIOK1	4	65582
86	Q9H3U1	UN45A	4	103076
87	Q9Y230	RUVB2	4	51156

Supplementary Table S3. Docking Score of PHF14-ePHD domain with DNA probes

Protein	Probe	Docking Score (Mean±SD)
PHF14-ePHD WT	unmethylated CG-rich	-184.1±2.93
	methylated CG-rich	-165.4±1.16
	AT-rich	-163.9±4.72
PHF14-ePHD MT	unmethylated CG-rich	-154.4±2.60
	methylated CG-rich	-155.2±1.90
	AT-rich	-153.8±5.26

Note: CG-rich probe: sense, 5’- ACGGCTCGTCGCTACGGCTCGTCGCT -3’, antisense, 5’- AGCGACGA GCCGTAGCGACGAGCCGT -3’; AT-rich probe: sense, 5’- CATTAGATGATAGCATTAGATGATAG -3’, antisense, 5’- CTATCATCTAATGCTATCATCTAATG -3’, in which the positions of methyl-C are underlined.

Supplementary Table S4. Patients' information in ctDNA analysis

Patients ID	M status	Survival time (months)	Survival status	SMAD7 %CG methylation in ctDNA before surgery	SMAD7 %CG methylation in ctDNA after surgery
P1	non-met	60	0	0.383	0.325
P2	non-met	60	0	0.297	0.143
P3	non-met	60	0	0.535	0.382
P4	non-met	60	0	0.257	0.176
P5	non-met	60	0	0.584	0.217
P6	non-met	60	0	0.313	0.242
P7	non-met	60	0	0.436	0.353
P8	non-met	60	0	0.275	0.065
P9	non-met	60	0	0.474	0.185
P10	non-met	60	0	0.368	0.333
P11	non-met	57.63	1	0.594	0.314
P12	non-met	43.25	1	0.527	0.285
P13	non-met	60	0	0.336	0.083
P14	non-met	60	0	0.489	0.302
P15	non-met	36.82	1	0.443	NA
P16	non-met	60	0	0.396	NA
P17	non-met	NA	NA	0.313	NA
P18	non-met	NA	NA	0.257	NA
P19	non-met	60	0	0.598	NA
P20	met	2.19	1	0.866	NA
P21	met	60	0	0.794	NA
P22	met	37.26	1	0.743	NA
P23	met	27.49	1	0.696	NA
P24	met	6.49	1	0.882	NA
P25	met	NA	NA	0.536	NA
P26	met	60	0	0.634	NA
P27	met	29.43	1	0.751	NA
P28	met	60	0	0.826	NA
P29	met	57.98	1	0.485	NA
P30	met	49.29	1	0.648	NA
P31	met	NA	NA	0.519	NA

P32	met	52.46	1	0.454	NA
P33	met	21.23	1	0.856	NA
P34	met	5.39	1	0.772	NA
P35	met	18.53	1	0.653	NA
P36	met	54.88	1	0.621	NA
P37	met	44.53	1	0.586	NA
P38	met	11.21	1	0.554	NA
P39	met	18.81	1	0.828	NA
P40	met	NA	NA	0.497	NA
P41	met	23.32	1	0.519	NA
P42	met	60	0	0.633	NA
P43	met	60	0	0.719	NA
P44	met	13.34	1	0.887	NA
P45	met	30.49	1	0.553	NA
P46	met	33.21	1	0.494	NA
N1	NA	NA	NA	0.313	NA
N2	NA	NA	NA	0.244	NA
N3	NA	NA	NA	0.416	NA
N4	NA	NA	NA	0.198	NA
N5	NA	NA	NA	0.123	NA
N6	NA	NA	NA	0.326	NA
N7	NA	NA	NA	0.224	NA
N8	NA	NA	NA	0.398	NA
N9	NA	NA	NA	0.124	NA
N10	NA	NA	NA	0.206	NA
N11	NA	NA	NA	0.166	NA
N12	NA	NA	NA	0.079	NA
